# On the Identification of Noise Covariances and Adaptive Kalman Filtering: A New Look at a 50 Year-Old Problem

**DOI:** 10.1109/access.2020.2982407

**Published:** 2020-03-23

**Authors:** LINGYI ZHANG, DAVID SIDOTI, ADAM BIENKOWSKI, KRISHNA R. PATTIPATI, YAAKOV BAR-SHALOM, DAVID L. KLEINMAN

**Affiliations:** 1 Department of Electrical and Computer Engineering, University of Connecticut, Storrs, CT 06269, USA; 2 U.S. Naval Research Laboratory, Marine Meteorology Division, Monterey, CA 93943, USA

**Keywords:** Adaptive filtering, Kalman filter, minimal polynomial, noise covariance estimation, adaptive gradient descent

## Abstract

The Kalman filter requires knowledge of the noise statistics; however, the noise covariances are generally *unknown*. Although this problem has a long history, reliable algorithms for their estimation are scant, and necessary and sufficient conditions for identifiability of the covariances are in dispute. We address both of these issues in this paper. We first present the necessary and sufficient condition for unknown noise covariance estimation; these conditions are related to the rank of a matrix involving the auto and cross-covariances of a weighted sum of innovations, where the weights are the coefficients of the minimal polynomial of the closed-loop system transition matrix of a stable, but not necessarily optimal, Kalman filter. We present an optimization criterion and a novel six-step approach based on a successive approximation, coupled with a gradient algorithm with adaptive step sizes, to estimate the steady-state Kalman filter gain, the unknown noise covariance matrices, as well as the state prediction (and updated) error covariance matrix. Our approach enforces the structural assumptions on unknown noise covariances and ensures symmetry and positive definiteness of the estimated covariance matrices. We provide several approaches to estimate the unknown measurement noise covariance *R* via *post-fit residuals*, an approach not yet exploited in the literature. The validation of the proposed method on five different test cases from the literature demonstrates that the proposed method significantly outperforms previous state-of-the-art methods. It also offers a number of novel machine learning motivated approaches, such as sequential (one sample at a time) and mini-batch-based methods, to speed up the computations.

## I. INTRODUCTION

The Kalman filter (KF) [23] is the optimal state estimator for linear dynamic systems driven by Gaussian white noise with measurements corrupted by Gaussian white noise.^1^ In the classical design of a Kalman filter, the noise covariance matrices are assumed known and they, along with the system dynamics, determine the achievable filter’s accuracy. However, in many practical situations, including noisy feature data in machine learning, the statistics of the noise covariances are often unknown or only *partially* known. Thus, noise identification is an essential part of adaptive filtering. Adaptive filtering has numerous applications in engineering [36], machine learning [9], econometrics [11], weather forecasting [10], [20], [35], [46], to name a few.

We were motivated by the following learning problem: Given a vector time series and a library of models of system dynamics for the data (e.g., a Wiener process, a white noise acceleration model, also called nearly constant velocity model, or a white noise jerk model, also called nearly constant acceleration model), find a suitable process noise and measurement noise model and the best system dynamics for the time series. The problem we consider in this paper is limited to finding a suitable process noise and measurement noise covariance for a given dynamic model.

### A. PREVIOUS WORK

The approaches for estimating the noise covariance matrices for a Kalman filter can be broadly classified into four general categories: Bayesian inference, maximum likelihood estimation, covariance-matching, and correlation methods. The first two categories pose the noise covariance estimation problem as a parameter estimation problem.

In the Bayesian inference approach [19], the covariance estimation problem is solved by obtaining the posterior probability density function (pdf) of the unknown parameters (in this case, the noise covariance matrix elements) from their prior pdf and the observed measurements using the Bayes’ formula recursively. In 2013, Matisko and Havlena [32] proposed a new Bayesian method to estimate the unknown covariance matrices. They first use a Monte Carlo method to generate a grid of possible unknown covariance matrix pairs (*Q, R*) with more density near the highest prior probability. Then, they compute the likelihood and posterior probability after performing state estimation for each pair using a Kalman filter. In general, the Bayesian approach suffers from the curse of dimensionality and is computationally intractable due to the fact that it involves numerical integration or Monte Carlo simulations over a very large parameter space.

In maximum likelihood estimation [25], [48], the noise statistics are obtained by maximizing the probability density function of the measurement residuals generated by the filter, which is the likelihood of the filter parameters [2]. These filter-based maximum likelihood methods require nonlinear programming based optimization and are computationally intractable. Shumway and Stoffer [47] utilize the expectation maximization (EM) algorithm [12], which requires the smoothed estimates of the system state. This approach starts with the smoothed estimation of the system state given an estimate of the initial state and noise covariance matrices. Then, the unknown parameters are estimated via maximum likelihood estimation using the smoothed state estimates obtained from the expectation step. Later, Ghahramani and Hinton [17] present an extension of [47] that can account for an unknown observation matrix in linear dynamic systems. They then go on to use forward and backward recursions to estimate the noise covariance matrices. This process is repeated until the estimated parameters converge. In addition to computational complexity, this method suffers from convergence to a local optimum.

The basic idea of the covariance-matching techniques [38] is that the sample covariance of the innovations should be consistent with its theoretical value. In [38], the unknown noise covariances are estimated from the sample covariance computed from the innovation sequences accumulated over the entire historical data (or in a moving time window). In this method, if the estimated innovation covariance value is much larger than the theoretical value, then the process noise covariance is increased. The convergence has never been proved for this method.

With regard to correlation methods, Heffes [18] derived an expression for the covariance of the state error and of the innovations of any suboptimal filter as a function of noise covariances. This expression serves as a fundamental building block in the correlation methods. The first innovation-based technique to estimate the optimal Kalman filter gain and the unknown noise covariance matrices via the correlations of innovations from an arbitrary initial stabilizing filter gain was introduced by Mehra [33]. Another procedure to carry out the identification of unknown optimal Kalman filter gain and the noise covariance matrices is by Carew and Bélanger [7]. Their strategy calculates the Kalman filter gain based on the estimation error that is defined as the discrepancy between the optimal state estimates obtained from the optimal Kalman filter gain and the state estimates obtained from an arbitrary suboptimal Kalman filter gain. There is a question as to whether the correlation method is sensitive to the initial Kalman filter gain selection. Mehra suggested to repeat the noise covariance estimation steps with the obtained gain from the first attempt to improve the estimation. However, Carew and Bélanger [7] claim that if the optimal Kalman filter gain is used as the initial condition, then the approximations in Mehra’s approach are such that the correctness of the optimal gain will not be confirmed.

Later, Neethling and Young [39] suggested to combine the noise covariance matrices in a vector and solve a single least squares or weighted least squares problem to improve the performance of Mehra and Carew-Bélanger’s approaches. In 2006, Odelson *et al.* [41], [42] developed the autocovariance least squares method to estimate the noise covariance matrices by applying the suggestions of [39] on Mehra’s approach and using the Kronecker operator. The algorithm defines a multistep autocovariance function between the measurements, which is used to develop a linear least squares formulation to estimate the noise covariance matrices. Duník *et al.* [14] compared the method presented by Odelson *et al.* [42] to a combined state and parameter estimation approach.

An interesting variant of the correlation methods is to utilize the output correlations. In 1972, Mehra [34] proposed an output correlation technique to directly estimate the optimal Kalman filter gain. This method has the advantage of being non-recursive compared to the innovation correlation techniques. However, the poor estimates of sample output correlation functions can lead to an ill-conditioned Riccati equation.

The contributions of the present paper are as follows:

1. A necessary and sufficient condition for the identifiability of unknown noise covariances is provided for a Gauss-Markov system. This involves the rank of the auto and cross-covariances of the weighted sum of innovations of a suboptimal filter, where the weights are the coefficients of the minimal polynomial of the state transition matrix.
2. A novel six-step solution approach via a successive approximation and adaptive gradient descent scheme with a new objective function to obtain the unknown noise covariance matrices *Q* and *R*, as well as the steady-state Kalman filter gain *W*, and the steady-state state prediction covariance matrix $\bar{P}$ or the updated state covariance matrix *P*, is proposed. This ensures positive definite *Q* and positive definite *R*, as well as $\bar{P}$ and *P*.
3. Several novel approaches to estimate the unknown noise covariance matrix *R* are derived via utilization of the *post-fit residual*, which has not yet been discussed in the literature.
4. Convergence proofs in [7] assumed that time averages are the same as ensemble averages. This is only approximate with finite data. Consequently, these methods either diverge or result in largely inaccurate estimates of unknown covariances.
5. Our approach can enforce structural assumptions on *Q* and *R* (e.g., diagonality of *Q* and *R*, symmetry and positive definiteness).

The paper is organized as follows. In Section II, we provide an overview of the Kalman filter and derive a new Riccati equation for the updated state covariance. Then, in Section III, we discuss the necessary and sufficient condition for the unknown noise covariances’ estimation. We briefly discuss different approaches to obtaining the unknown covariance parameters in Section IV. Then, in Section V, we discuss a convergent version of Mehra’s method to estimate the optimal Kalman filter gain. In Section VI, we derive five different ways to obtain *R*. Section VII provides a method to estimate the process noise covariance matrix *Q* and the steady-state updated state covariance *P*, iteratively. All these methods are combined in Section VIII, where we present a systematic process to find the optimal filter gain *W*, the innovation covariance *S*, the measurement noise covariance *R*, the steady-state state prediction (or updated state) covariance $\bar{P}$ (*P*) and the process noise covariance *Q*. In Section IX, we specialize the approach to estimate *W*, *R*, *Q* and *P* for a process, where all the states are observed and for a Wiener process. Lastly, we apply our approach to five numerical examples from the literature in Section X, and conclude the paper in Section XI. In this paper, all the subscripts denote matrix indices. The iteration variable is superscript with (·) to differentiate the notation from exponents.

## II. PLANT AND MEASUREMENT MODEL FOR THE KALMAN FILTER

The notation used in the remainder of this paper is listed in Table 1. Consider the discrete-time linear dynamic system

(1) x(k+1)=Fx(k)+Γv(k)

(2) z(k)=Hx(k)+w(k)

(i.e., a Gauss-Markov system) where *x*(*k*) is an *n_x_*-dimensional state vector, *F* is the state transition matrix of the system, *H* is the *n_z_* × *n_x_* measurement matrix, and Γ is the *n_x_* × *n_v_* dimensional noise gain matrix. The sequences *v*(*k*), *k* = 0, 1, …, and *w*(*k*), *k* = 0, 1, …, are zero-mean white Gaussian noises with covariance matrices *Q* and *R*, respectively. The two noise sequences and the initial state are assumed to be mutually independent. The matrices *Q* and *R* are assumed to be positive definite. Note that even if *Q* is positive definite, Γ*Q*Γ′ need not be; it can be positive semi-definite. We assume that the system is observable and (*F*, Γ*Q*^1/2^) is controllable.^2^

Given the estimate $\hat{x}(k|k)$, the Kalman filter [2], [23] estimates the state at the next time instant *k* + 1 as

(3) $$\hat{x}(k+1|k)=F\hat{x}(k|k)$$

(4) $$v(k+1)=z(k+1)-H\hat{x}(k+1|k)$$

(5) $$\hat{x}(k+1|k+1)=\hat{x}(k+1|k)\;\;+W(k+1)v(k+1)$$

(6) $$P(k+1|k)=FP(k|k)F^{\prime }+\Gamma Q\Gamma^{\prime }$$

(7) $$S(k+1)=HP(k+1|k)H^{\prime }+R$$

(8) $$W(k+1)=P(k+1|k)H^{\prime }S(k+1{)}^{-1}$$

(9) $$P(k+1|k+1)=P(k+1|k)\;\;-W(k+1)S(k+1)W(k+1{)}^{\prime }$$

where the estimate $\hat{x}(k+1|k)$ is the one-step extrapolated estimate of the state vector *x*(*k*) based on the measurements up to *k*, *W*(*k*), *k* = 1, …, *N* is the sequence of Kalman filter gains, *ν*(*k*), *k* = 1, …, *N* is the innovation sequence, *P*(*k* + 1∣*k*) is the state prediction covariance, *S*(*k* + 1) is the measurement prediction (or innovation) covariance, and *P*(*k* + 1∣*k* + 1) is the updated state error covariance.

The six-step approach in this paper is designed specifically for a steady-state Kalman filter. The steady-state state prediction covariance matrix $\bar{P}$ satisfies an algebraic Riccati equation.

(10) $$\bar{P}=F[\bar{P}-\bar{P}H^{\prime }(H\bar{P}H^{\prime }+R{)}^{-1}H\bar{P}]F^{\prime }+\Gamma Q\Gamma^{\prime }$$

The steady-state updated state covariance, denoted as *P*, can also be computed via another algebraic Riccati equation (see Appendix A).

(11) $$P=FPF^{\prime }-PH^{\prime }(R-HPH^{\prime }{)}^{-1}HP^{\prime }+\Gamma Q\Gamma^{\prime }$$

Evidently,

(12) $$P=\bar{P}-WSW^{\prime }$$

(13) $$=(I_{n_{x}}-WH)\bar{P}(I_{n_{x}}-WH{)}^{\prime }+WRW^{\prime }$$

where (13) is known as the Joseph form; *W* and *S* are the steady-state optimal gain, and the steady-state innovation covariance, respectively, and are given by

(14) $$W=\bar{P}H^{\prime }S^{-1}\;\;=\bar{P}H^{\prime }(H\bar{P}H^{\prime }+R{)}^{-1}\;\;=PH^{\prime }R^{-1}$$

(15) $$S=E[v(k)v(k{)}^{\prime }]=H\bar{P}H^{\prime }+R$$

Note that (*I*_*n*_*x*__ − *WH*) is invertible, but need not be stable (i.e., eigenvalues need not be inside the unit circle).

## III. IDENTIFIABILITY OF *Q* AND *R*

One major issue in the previous literature involves the necessary conditions to estimate the unknown covariance matrices. Mehra [33] claimed that the system must be observable and controllable; however, Odelson [42] provided a counter-example wherein the system was observable and controllable, but the full *Q* matrix was not estimable. Following the ideas in [49], we prove that the necessary and sufficient condition (as detailed in Appendix B) to estimate the unknown covariance matrices in a system is *directly related* to its minimal polynomial of

(16) $$\bar{F}=F(I_{n_{x}}-WH),$$

its stable closed-loop filter matrix $\bar{F}$, and a transformation of the innovations based on the coefficients of the minimal polynomial. Let us define $\tilde{x}(k+1|k)$ to be the predicted error between the state *x*(*k* + 1) and its predicted state $\hat{x}(k+1|k)$, that is,

(17) $$\tilde{x}(k+1|k)=x(k+1)-\hat{x}(k+1|k)$$

We can rewrite $\hat{x}(k+1|k)$ in terms of $\tilde{x}$, that is,

(18) $$\hat{x}(k+1|k)=F\hat{x}(k|k-1)+FWH\tilde{x}(k|k-1)+FWw(k)$$

Then, substituting (18) into (17) and using (1), we have

(19) $$\tilde{x}(k+1|k)=\bar{F}\tilde{x}(k|k-1)+\Gamma \;v(k)-FWw(k)$$

where $\bar{F}$ is defined in (16). We can also write *ν*(*k*) in terms of $\tilde{x}$, that is

(20) $$v(k)=H\tilde{x}(k|k-1)+w(k)$$

Let us define the *m^th^* order minimal polynomial of $\bar{F}$ as

(21) $$\sum_{i=0}^{m}a_{i}{\bar{F}}^{m-i}=0;\;\;a_{0}=1$$

(22) $$v(k)=H\;{\bar{F}}^{m}\tilde{x}(k-m|k-m-1)+\left\{H\sum_{j=0}^{m-1}{\bar{F}}^{m-1-j}[\Gamma \;v(k-m+j)-FWw(k-m+j)]\right\}+w(k)$$

(23) $$\xi (k)=\sum_{i=0}^{m}a_{i}v(k-i)$$

(24) $$=\sum_{i=0}^{m}a_{i}\left[H\left\{\sum_{j=0}^{m-i-1}{\bar{F}}^{m-i-1-j}\;[\Gamma \;v(k-m+j)-FWw(k-m+j)]\right\}+w(k-i)\right]$$

(25) $$=\sum_{i=0}^{m}a_{i}\left[H\left\{\sum_{l=i+1}^{m}{\bar{F}}^{l-i-1}\;[\Gamma v(k-l)-FWw(k-l)]\right\}+w(k-i)\right]$$

(26) $$=\sum_{l=1}^{m}H\left(\sum_{i=0}^{l-1}a_{i}{\bar{F}}^{l-i-1}\right)[\Gamma \;v(k-l)-FWw(k-l)]+\sum_{l=0}^{m}a_{l}w(k-l)$$

(27) $$=\sum_{l=1}^{m}B_{l}v(k-l)+\sum_{l=0}^{m}G_{l}w(k-l)$$

Then, the innovations *ν*(*k*) can be written as (22), shown at the bottom of the next page.

Note that we apply the minimal polynomial of $\bar{F}$ to ensure that the innovation in (22), is stationary. Let us define *ξ*(*k*) as (23)-(27), shown at the bottom of the next page, where $B_{l}$ and $G_{l}$ are the sum of two moving average processes driven by the process noise and the measurement noise, that is,

(28) $$B_{l}=H\left(\sum_{i=0}^{l-1}a_{i}{\bar{F}}^{l-i-1}\right)\Gamma$$

(29) $$G_{l}=\left[a_{l}I_{n_{z}}-H\left(\sum_{i=0}^{l-1}a_{i}{\bar{F}}^{l-i-1}\right)FW\right]$$

(30) $$G_{0}=I_{n_{z}}$$

Denoting *L_j_* = *E* [*ξ*(*k*)*ξ*(*k* − *j*)′], for *j* = 0, 1, 2, …, *m*, we have

(31) $$L_{j}=\sum_{i=j+1}^{m}B_{i}QB_{i-j}^{\prime }+\sum_{i=j}^{m}G_{i}RG_{i-j}^{\prime }$$

(32) $$L_{j}=\sum_{l=1}^{n_{v}}\sum_{p=1}^{n_{v}}q_{lp}\left[\sum_{i=j+1}^{m}b_{i,l}b_{i-j,p}^{\prime }\right]+\sum_{l=1}^{n_{z}}\sum_{p=1}^{n_{z}}r_{lp}\left[\sum_{i=j}^{m}g_{i,l}g_{i-j,p}^{\prime }\right]$$

(33) $$=\sum_{l=1}^{n_{v}}\left\{\sum_{p=1}^{l}q_{lp}\left[\sum_{i=j+1}^{m}b_{i,l}b_{i-j,p}^{\prime }\right]+\sum_{p=l+1}^{m}q_{lp}\left[\sum_{i=j+1}^{m}b_{i,l}b_{i-j,p}^{\prime }\right]\right\}\;\;+\sum_{l=1}^{n_{z}}\left\{\sum_{p=1}^{l}r_{lp}\left[\sum_{i=j}^{m}g_{i,l}g_{i-j,p}^{\prime }\right]+\sum_{p=l+1}^{m}r_{lp}\left[\sum_{i=j}^{m}g_{i,l}g_{i-j,p}^{\prime }\right]\right\}$$

(34) $$=\sum_{l=1}^{n_{v}}\left\{q_{ll}\left[\sum_{i=j+1}^{m}b_{i,l}b_{i-j,l}^{\prime }\right]+\sum_{p=l+1}^{n_{v}}q_{lp}\left[\sum_{i=j+1}^{m}b_{i,l}b_{i-j,p}^{\prime }+b_{i,p}b_{i-j,l}^{\prime }\right]\right\}\;\;+\sum_{l=1}^{n_{z}}\left\{r_{ll}\left[\sum_{i=j}^{m}g_{i,l}g_{i-j,p}^{\prime }\right]+\sum_{p=l+1}^{n_{z}}r_{lp}\left[\sum_{i=j}^{m}g_{i,l}g_{i-j,p}^{\prime }+g_{i,p}g_{i-j,l}^{\prime }\right]\right\}$$

We know that *Q* = [*q_ij_*] is an *n_v_* × *n_v_* positive semi-definite and symmetric matrix, and *R* = [*r_ij_*] is an *n_z_* × *n_z_* positive definite and symmetric matrix. Utilizing the symmetry of *Q* and *R*, and letting *b_i,l_* and *g_i,l_* denote the *l*-th column of $B_{i}$ and $G_{i}$, respectively, we can rewrite (31) as (32)-(34) shown at the bottom of the next page.

From (34), we can form **the noise covariance identifiability matrix** I of dimension $\left(m+1)n_{z}^{2}\times \frac{1}{2}[n_{v}(n_{v}+1)+n_{z}(n_{z}+1)\right]$, as in Algorithm 1. Algorithm 1 uses the vec(*A*) function to convert a matrix *A* into a column vector. For a *p*×*n* matrix *A*,

(35) $$\text{vec}(A)≜[a_{11},\ldots ,a_{p1},a_{12},\ldots ,a_{p2},\ldots ,a_{1n},\ldots ,a_{pn}{]}^{\prime }$$

Using (34) and collecting terms corresponding to each *q_lp_*, *p* = *l*, *l* + 1, …, *n_v_* and each *r_lp_*, *p* = *l*, *l* + 1, …, *n_z_* into the corresponding columns of I, we obtain the following identifiability condition that must be satisfied by *Q* and *R*,

(36) $$I\left[\begin{array}{c} \text{vec}(Q)\\ \text{vec}(R) \end{array}\right]=\left[\begin{array}{c} L_{0}\\ L_{1}\\ \vdots \\ L_{m} \end{array}\right]$$

The linearity of (36) implies the full rank condition on I. Since *R* is always estimable because $G_{m}$ (recall that *m* is the order of minimal polynomial) is invertible,^3^ the maximum number of unknowns in *Q* that can be estimated must be less than or equal to the minimum number of independent measurements minus the number of unknowns in *R*.

**Table:** 

Algorithm 1 Construction of the Noise Covariance Identifiability Matrix I
$$\begin{array}{cc} \; & \;1:\mathrm{for}\;j≔0\;:m\;\mathrm{do}\\ \; & \;2:\;\;\;\;r=j*n_{z}^{2}\\ \; & \;3:\;\;\;\;k\leftarrow 0\\ \; & \;4:\;\;\;\;\mathrm{for}\;l≔1:n_{v}\;\mathrm{do}\\ \; & \;5:\;\;\;\;\;\;\;k\leftarrow k+1\\ \; & \;6:\;\;\;\;\;\;\;b=\sum_{i=j+1}^{m}\;[b_{i,l}b_{i-j,l}^{\prime }{]}^{\prime }\\ \; & \;7:\;\;\;\;\;\;\;I(r+1\;:\;r+n_{z}^{2},k)\leftarrow \text{vec}(b)\\ \; & \;8:\;\;\;\;\;\;\;\mathrm{for}\;p≔l+1:n_{v}\;\mathrm{do}\\ \; & \;9:\;\;\;\;\;\;\;\;\;\;k\leftarrow k+1\\ \; & 10:\;\;\;\;\;\;\;\;\;\;c_{j,l,i}(p)=[b_{i,l}b_{i-j,p}^{\prime }+b_{i,p}b_{i-j,l}^{\prime }{]}^{\prime }\\ \; & 11:\;\;\;\;\;\;\;\;\;\;d=\sum_{i=j+1}^{m}\;c_{j,l,i}(p)\\ \; & 12:\;\;\;\;\;\;\;\;\;\;I(r+1:r+n_{z}^{2},k)\leftarrow \text{vec(d)}\\ \; & 13:\;\;\;\;\;\;\;\mathrm{end}\;\mathrm{for}\\ \; & 14:\;\;\;\;\mathrm{end}\;\mathrm{for}\\ \; & 15:\;\;\;\;\mathrm{for}\;l≔1:n_{z}\;\mathrm{do}\\ \; & 16:\;\;\;\;\;\;\;k\leftarrow k+1\\ \; & 17:\;\;\;\;\;\;\;g=\sum_{i=j}^{m}\;[g_{i,l}g_{i-j,p}^{\prime }{]}^{\prime }\\ \; & 18:\;\;\;\;\;\;\;I(r+1:r+n_{z}^{2},k)\leftarrow \text{vec(g)}\\ \; & 19:\;\;\;\;\;\;\;\mathrm{for}\;p≔l+1:n_{z}\;\mathrm{do}\\ \; & 20:\;\;\;\;\;\;\;\;\;\;k\leftarrow k+1\\ \; & 21:\;\;\;\;\;\;\;\;\;\;h_{j,l,p}(i)=[g_{i,l}g_{i-j,p}^{\prime }+g_{i,p}g_{i-j,l}^{\prime }{]}^{\prime }\\ \; & 22:\;\;\;\;\;\;\;\;\;\;f=\sum_{i=j}^{m}\;h_{j,l,p}(i)\\ \; & 23:\;\;\;\;\;\;\;\;\;\;I(r+1:r+n_{z}^{2},k)\leftarrow \text{vec(f)}\\ \; & 24:\;\;\;\;\;\;\;\mathrm{end}\;\mathrm{for}\\ \; & 25:\;\;\;\;\mathrm{end}\;\mathrm{for}\\ \; & 26:\mathrm{end}\;\mathrm{for} \end{array}$$

That is

(37) $$\text{rank}(I)-n_{R}>n_{Q}$$

where *n_R_* is the number of unknowns in *R*, and *n_Q_* is the number of unknowns in *Q*

To illustrate the necessity and sufficiency of this condition, consider an example system from [42],

(38) $$x(k)=\left[\begin{array}{ccc} 0.9 & 0 & 0\\ 1 & 0.9 & 0\\ 0 & 0 & 0.9 \end{array}\right]x(k-1)+v(k-1)$$

(39) $$z(k)=\left[\begin{array}{ccc} 0 & 1 & 0\\ 0 & 0 & 1 \end{array}\right]x(k)+w(k)$$

with *Q* being a full 3 × 3 positive definite symmetric matrix and *R* being a full 2 × 2 positive definite symmetric matrix. Since the rank of I is not affected by *W* (the observability condition is independent of the filter gain matrix), one can examine the rank of I for *W* = 0 for convenience. In this case, the minimal polynomial coefficients are

(40) $$[a_{0}\;\;a_{1}\;\;a_{2}{]}^{\prime }=[1\;-1.8\;0.81{]}^{\prime }$$

The B and G matrices are

(41) $$B_{1}=\left[\begin{array}{ccc} 1 & \;1 & \;0\\ 0 & 0\; & 1\; \end{array}\right]B_{2}=\left[\begin{array}{ccc} 1 & -0.9\; & 0\;\\ 0 & \;0 & \;\;-0.9 \end{array}\right]$$

(42) $$G_{0}=\left[\begin{array}{cc} 1 & 0\\ 0 & 1 \end{array}\right]\;\;G_{1}=\left[\begin{array}{cc} -1.8 & 0\\ 0 & -1.8 \end{array}\right]$$

(43) $$G_{2}=\left[\begin{array}{cc} 0.81 & 0\\ 0 & 0.81 \end{array}\right]$$

Here, I is a 12 × 9 matrix with a rank of 8. Since there are 9 unknown variables (6 in *Q* and 3 in *R*), the covariance matrix elements are not identifiable. However, if *E*[*v*(*k*)*v*(*k*)′] is diagonal, as is typically the case, then the covariance matrix elements are identifiable because there are only 6 unknown variables (full *R* matrix and three diagonal elements of *Q*).

Another example to illustrate the necessity and sufficiency of this condition is to consider the system

(44) $$x(k)=\left[\begin{array}{cc} 0.1 & 0\\ 0 & 0.2 \end{array}\right]x(k-1)+\left[\begin{array}{cc} 1 & 0\\ 0 & 2 \end{array}\right]v(k)$$

(45) z(k)=[10]x(k)+w(k)

with *Q* being a diagonal 2×2 positive definite diagonal matrix and *R* being a scalar. Similarly, we examine the rank of I for *W* = 0 and obtain the minimal polynomial coefficients,

(46) $$[a_{0}\;\;a_{1}\;a_{2}{]}^{\prime }=[1\;\;-0.3\;\;0.02{]}^{\prime }$$

The B and G matrices are

(47) $$B_{1}=[1\;\;0]\;B_{2}=[-0.2\;\;0]$$

(48) $$G_{0}=1\;\;G_{1}=-0.3\;\;G_{2}=0.02$$

Here,

(49) $$I=\left[\begin{array}{ccc} 1.04 & 0 & 1.09\\ -0.2 & 0 & -0.31\\ 0 & 0 & 0.02 \end{array}\right]$$

has a rank of 2. Since there are 3 unknown variables (2 in *Q* and 1 in *R*), the covariance matrix elements are not identifiable.

Note that the minimal polynomial can be used to estimate the unknown covariances *R* and *Q* via quadratic programming techniques. Furthermore, it can be used to estimate the optimal gain *W*, as in [49] and Appendix D; however, reliable and accurate estimation of the parameters of vector moving average processes is still an unresolved problem [16], [24], [31], [45].

## IV. APPROACHES TO OBTAIN FILTER PARAMETERS

There are two competing approaches for the estimation of the filter parameters *W*, *R*, *Q*, and $\bar{P}$. The first approach is to estimate the noise covariance matrices first and subsequently the Kalman filter gain *W* and the predicted state covariance $\bar{P}$ are computed given the estimated noise covariance matrices [32], [48]. This method has an underlying problem in that it involves the sum of two moving average processes. Additionally, the autoregressive moving average (ARMA) approach, pioneered in the econometric literature, does not extend naturally to sums of moving average processes and we have found the resulting algorithms [16], [24], [31], [45] to have erratic computational behavior.

The second approach is to estimate the Kalman filter gain *W* from the measured data first [7], [33]. Given the optimal *W*, we can compute *R*, *Q* and $\bar{P}$ (this approach is applied in this paper). The proposed *R*, *Q* and $\bar{P}$ estimates in this paper are valid as long as an optimal gain *W* is provided. There are many ways to obtain the optimal Kalman filter gain *W*. The techniques listed in this paper to obtain the optimal *W*, that is, Section V and Appendix D, are by no means all-inclusive, and several such methods may be suitable for a given problem. For example, the optimal gain *W* can be obtained from the suboptimal Kalman filter residual [8], solving the minimal polynomial problem [49], utilizing the least squares method on the observable form [6], and utilizing a second Kalman filter to track the error in the estimated residual of the first Kalman filter [44], to name a few.

## V. ESTIMATION OF *W*

This section includes the discussion of two different approaches to estimate the optimal Kalman filter gain *W*, namely, the minimal polynomial approach and the successive approximation, coupled with an adaptive gradient descent scheme, on a criterion based on innovation correlations. The derivation of the minimal polynomial approach is detailed in Appendix D. This approach assumes the system to be purely driven by the optimal innovation. In doing so, the estimation of the optimal Kalman gain can be achieved via a vector auto-regressive model approximation of a vector moving average process. However, from limited testing on examples chosen in this paper, this approach was found to be numerically unstable, only performing well on systems with no eigenvalues close to unity. In fact, the vector auto-regressive model has various numerical problems and an accurate and reliable algorithm to obtain the solution still remains to be developed [24]. Therefore, we omit this approach from the paper and focus on minimization of the innovation correlations using a successive approximation and adaptive gradient descent method.

In the sequel, we describe in detail the approach of our paper using the correlation-based criterion. If the Kalman filter gain *W* is not optimal, the innovation sequence $\{v(k){\}}_{k=1}^{N}$ is correlated. We can use the innovation sequence of any stable suboptimal Kalman filter and compute *M* sample covariance matrices, as in [33]:

(50) $$\hat{C}(i)=\frac{1}{N-M}\sum_{j=1}^{N-M}v(j)v(j+i{)}^{\prime }\;\;i=0,1,2,\ldots ,M-1$$

We know that the optimal Kalman filter gain *W* makes the autocorrelation function $\hat{C}(i)$, *i* = 0, 1, 2, …, *M* − 1 vanish for all *i* ≠ 0. Given the correlation matrix for *i* ≥ 1 as in [33], that is

(51) $$C(i)=E[v(k)v(k-i{)}^{\prime }]=H\;{\bar{F}}^{i-1}F\;[\bar{P}H^{\prime }-WC(0)]$$

where $\bar{F}$ is as in (16). We define the objective function *J* to be minimized as

(52) J=12tr{∑i=1M−1[diag(C^(0))]−12C^(i)′}×{[diag(C^(0))]−1C^(i)[diag(C^(0))]−12}

where diag(*C*) is the Hadamard product of an identity matrix, of same dimension as *C*, with *C*

(53) diag(C)=I⊙C

This objective function is selected to minimize the sum of the normalized $\hat{C}(i)$ with respect to the corresponding diagonal elements of $\hat{C}(0)$ for *i* > 0. The optimal *J* becomes 0 as the sample size *N* tends to ∞ because the time averages are the same as ensemble averages given infinite data. Substituting (51) into (52) and utilizing the cyclic property of trace, we have

(54) $$J=\frac{1}{2}\text{tr}\left\{\sum_{i=1}^{M-1}\Theta (i)XE^{2}X^{\prime }\right\}$$

where

(55) $$\Theta (i)=\Phi (i{)}^{\prime }E^{2}\Phi (i)$$

(56) $$\Phi (i)=H{\bar{F}}^{i-1}F$$

(57) X=Ψ−WC(0)

(58) $$\Psi =\bar{P}H^{\prime }$$

(59) $$E={\left[\text{diag}\;(C(0))\right]}^{-\frac{1}{2}}$$

For ill-conditioned systems, a regularization term *λ*_*W*_tr(*WW*′) can be added to convexify the objective function. Taking the gradient^4^ of (54) with respect to *W*, we get

(60) $$\nabla wJ=-\sum_{i=1}^{M-1}\Phi (i{)}^{\prime }E^{2}C(i)E^{2}C(0)-F^{\prime }ZFX\;\;-\sum_{\ell =0}^{i-2}{\left[C(\ell +1)E^{2}C(i{)}^{\prime }E^{2}H{\bar{F}}^{i-\ell -2}\right]}^{\prime }$$

and *Z* is given by the Lyapunov equation

(61) $$Z={\bar{F}}^{\prime }Z\bar{F}+\frac{1}{2}\sum_{i}^{M-1}\Phi (i{)}^{\prime }E^{2}\hat{C}(i)E^{2}H+{\left(\Phi (i{)}^{\prime }E^{2}\hat{C}(i)E^{2}H\right)}^{\prime }$$

and *X* is obtained by rewriting (51) as

(62) $$\left[\begin{array}{c} HF\\ H\bar{F}F\\ \vdots \\ H{\bar{F}}^{M-1}F \end{array}\right]X=\left[\begin{array}{c} \hat{C}(1)\\ \hat{C}(2)\\ \vdots \\ \hat{C}(M-1) \end{array}\right]$$

Then, we can obtain *X* as

(63) $$X={\left[\begin{array}{c} HF\\ H\bar{F}F\\ \vdots \\ H{\bar{F}}^{M-1}F \end{array}\right]}^{†}\left[\begin{array}{c} \hat{C}(1)\\ \hat{C}(2)\\ \vdots \\ \hat{C}(M-1) \end{array}\right]$$

where *A*^†^ is the pseudoinverse of *A*, defined by

(64) $$A^{†}=(A^{\prime }A{)}^{-1}A^{\prime }$$

which exists, since we assume the system to be completely observable and *M* ≥ *n_x_*. The gradient direction can be used to obtain the optimal Kalman filter gain *W* iteratively through the bold driver method in [3], [29], [53]. Details of this application can be found in Section VIII-C2.

## VI. ESTIMATION OF *R*

### A. GENERAL R

Given the steady-state optimal gain *W* and the innovation covariance *S*, whose estimation is explained later in Section VIII, let *μ*(*k*), *k* = 1, …, *N* be the sequence of post-fit residuals of the Kalman filter, that is,

(65) $$\mu (k)=z(k)-H\hat{x}(k|k)$$

(66) $$=(I_{n_{z}}-HW)v(k)$$

Note that (*I*_*n*_*z*__ − *HW*) is invertible (rank *n_z_*) because (*I*_*n*_*z*__−*HW*) = *RS*^−1^ (proven below) and due to the assumption that *R* is positive definite.

*Proposition 1:* Given the optimal steady-state Kalman filter gain *W*, the post-fit residual sequence *μ*(*k*), and the innovation sequence *ν*(*k*), the joint covariance of these two sequences is

(67) $$\text{Cov}\left(\left[\begin{array}{c} v(k)\\ \mu (k) \end{array}\right]\right)=\left[\begin{array}{cc} S & R\\ R & R-HPH^{\prime } \end{array}\right]$$

*Proof:* On the right hand side of (67), the (1,1) block is simply the definition of the innovation covariance matrix in (15). Using (66), the (1,2) block in (67) is, given by

(68) $$E[\mu (k)v(k{)}^{\prime }]=(I_{n_{z}}-HW)E[v(k)v(k{)}^{\prime }]\;\;=(I_{n_{z}}-HW)S$$

Using (7) and (8),

(69a) $$E[\mu (k)v(k{)}^{\prime }]=(I_{n_{z}}-H\bar{P}H^{\prime }S^{-1})S$$

(69b) $$=S-H\bar{P}H^{\prime }=R$$

The (2,2) block of (67) is obtained as follows.

(70) $$G=E[\mu (k)\mu (k{)}^{\prime }]$$

(71) =E{[(Inz−HW)v(k)]}{[(Inz−HW)v(k)]′}

(72) $$=(I_{n_{z}}-HW)S(I_{n_{z}}-HW{)}^{\prime }$$

(73) $$=R(I_{n_{z}}-HW{)}^{\prime }=R-RW^{\prime }H^{\prime }$$

which, given (14), simplifies to

(74) $$G=R-HPH^{\prime }$$

Note that by using the Schur determinant identity [5], [51], the determinant of (67) is

(75) $$|\begin{array}{cc} S & R\\ R & R-HPH^{\prime } \end{array}|=|S||G-RS^{-1}R|=0$$

where the relationship *G* = *R* − *HPH*′ = *RS*^−1^*R* is proved in (74) and Proposition 2 below.

*Proposition 2:* Given the optimal steady-state Kalman filter gain *W* and the corresponding post-fit residual *μ*(*k*) and innovation *ν*(*k*) sequences, the covariance matrix *R* can be computed in the following five ways:

(76) $$R1\;:R=(I_{n_{z}}-HW)S$$

(77) $$R2\;:R=\frac{1}{2}\;\{E[\mu (k)v(k{)}^{\prime }+E[v(k)\mu (k{)}^{\prime }]\}$$

(78) $$R3\;:\text{Obtain}\;R\;\text{from}\;\;G=RS^{-1}R$$

(79) $$R4\;:R=\frac{1}{2}\;[G+S-HWSW^{\prime }H^{\prime }]$$

(80) $$R5\;:R=\frac{1}{2}\left\{G(I_{n_{z}}-W^{\prime }H^{\prime }{)}^{-1}+(I_{n_{z}}-HW{)}^{-1}G\right\}$$

*Proof:* **R1** is proven in (68). Method **R2** to estimate *R* is by symmetrizing (68). For method **R3** to estimate *R*, we can substitute (8) in (74) and rewrite *G* as

(81) $$G=R-H\bar{P}H^{\prime }+HWSW^{\prime }H^{\prime }$$

Then, by substituting (14) into (81)

(82) $$G=R-H\bar{P}H^{\prime }+H\bar{P}H^{\prime }S^{-1}H\bar{P}H^{\prime }$$

We also know from (15)

(83) $$(S-R)=H\bar{P}H^{\prime }$$

By substituting (83) into (82), we can write *G*, defined in (71), as

(84) $$G=R-(S-R)+(S-R)S^{-1}(S-R)$$

(85) $$=RS^{-1}R$$

(86) $$S=RG^{-1}R$$

Note that (85) is a continuous-time algebraic Riccati equation.^5^ Therefore, we can estimate *R* by solving the continuous-time Riccati equation, as in [1], or Kleinman’s method [27]. Some additional methods to solve the continuous-time algebraic Riccati equation can be found in [30]. We can also interpret (78) in terms of a Linear Quadratic Regulator (LQR) optimal control problem, where we can obtain *R* as the solution of the continuous-time algebraic Riccati equation associated with the optimal gain in the LQR problem. The computation of *R* is also related to the simultaneous diagonalization problem^6^ in linear algebra [51]. Note that, in the scalar case, *R* is the geometric mean of the variance of the post-fit residual and the innovation, as in the (1,2) block of (67).

For **R4**, we substitute (83) into (81) and rewrite *G* as

(87) $$G=R-(S-R)+HWSW^{\prime }H^{\prime }$$

(88) $$=2R-S+HWSW^{\prime }H^{\prime }$$

Solving for *R*, we obtain

(89) $$R=\frac{1}{2}\;\{G+S-HWSW^{\prime }H^{\prime }\}$$

thus, proving **R4**.

For **R5**, recall (68). We can rewrite (72) as

(90) $$G=(I_{n_{z}}-HW)S(I_{n_{z}}-HW{)}^{\prime }$$

(91) $$=R(I_{n_{z}}-HW{)}^{\prime }=(I_{n_{z}}-HW)R$$

Thus, we can compute *R* as

(92) $$\hat{R}=(I_{n_{z}}-HW{)}^{-1}\;G$$

(93) $$=G(I_{n_{z}}-W^{\prime }H^{\prime }{)}^{-1}$$

We can symmetrize the estimate of *R* by

(94) $$\hat{\hat{R}}=\frac{1}{2}\left\{G\;(I_{n_{z}}-W^{\prime }H^{\prime }{)}^{-1}+(I_{n_{z}}-HW{)}^{-1}\;G\right\}$$

proving **R5**.

Note that **R1–R5** are theoretically the same; however, they are numerically different. We recommend **R3**, since it ensures the positive definiteness of *R*.

### B. DIAGONAL R

When *R* is diagonal, we solve the least squares problem of

(95) $$\underset{X\geq 0}{\text{min}}\|X-R{\|}_{F}^{2}$$

where F, indicates the Frobenius norm. The positive definite *R* can be estimated from **R3**, given in Proposition 2. The solution is simply the diagonal elements of the estimated *R* from **R3**. This can also be interpreted as the masking operation to impose structural constraints on *R*, as discussed in the context of the estimation of *Q* in Section VII.

### C. USE OF SMOOTHED STATE ESTIMATE WITH ONE-STEP-LAG POST-FIT RESIDUALS

Note that *R* can also be estimated using one-step-lag smoothing on the post-fit residuals. Let us define the one-step-lag smoothed residual *s*(*k*) as in [37], that is,

(96) $$s(k)=z(k)-H\hat{x}(k|k+1)$$

(97) $$=z(k)-H\hat{x}(k|k)-HW_{1}v(k+1)$$

(98) $$W_{1}=\bar{P}{\tilde{F}}^{\prime }{\bar{P}}^{-1}W$$

where $\tilde{F}$ is defined as

(99) $$\tilde{F}=(I_{n_{x}}-WH)F=F^{-1}\bar{F}F$$

From (65), we can also write *s*(*k*) as a one-step moving average process

(100) $$s(k)=\mu (k)-HW_{1}v(k+1)$$

(101) $$=(I_{n_{z}}-HW)v(k)-HW_{1}v(k+1)$$

Therefore,

(102) $$E[s(k)v(k{)}^{\prime }]=(I_{n_{z}}-HW)C(0)-HW_{1}C(1{)}^{\prime }$$

and for the optimal Kalman filter gain *W*, we can write (102) as

(103) $$E[s(k)v(k{)}^{\prime }]=(I_{n_{z}}-HW)S=R$$

A similar expression can be derived for *E*[*s*(*k*)*μ*(*k*)′], that is,

(104) $$E[s(k)\mu (k{)}^{\prime }]=(I_{n_{z}}-HW)C(0)(I_{n_{z}}-HW{)}^{\prime }\;\;-HW_{1}C(1{)}^{\prime }(I_{n_{z}}-HW{)}^{\prime }$$

and for the optimal Kalman filter gain *W*, we have

(105) $$E[s(k)\mu (k{)}^{\prime }]=(I_{n_{z}}-HW)S(I_{n_{z}}-HW{)}^{\prime }\;\;=RS^{-1}R=G$$

Lastly, the expression for *E*[*s*(*k*)*s*(*k*)′] is

(106) $$E[s(k)s(k{)}^{\prime }]=(I_{n_{z}}-HW)C(0)(I_{n_{z}}-HW{)}^{\prime }\;\;+HW_{1}C(0)W_{1}^{\prime }H^{\prime }\;\;-(I_{n_{z}}-HW)C(1)(W_{1}{)}^{\prime }H^{\prime }\;\;-HW_{1}C(1{)}^{\prime }(I_{n_{z}}-HW{)}^{\prime }$$

(107) $$E[s(k)s(k{)}^{\prime }]=RS^{-1}R+H\bar{P}{\tilde{F}}^{\prime }{\bar{P}}^{-1}WSW^{\prime }{\bar{P}}^{-1}WSW^{\prime }{\bar{P}}^{-1}\tilde{F}\bar{P}H^{\prime }$$

(108) $$=RS^{-1}R+RW^{\prime }F^{\prime }(I_{n_{x}}-H^{\prime }W^{\prime })H^{\prime }R^{-1}SR^{-1}H(I_{n_{x}}-WH)FWR$$

(109) $$=RS^{-1}R+RW^{\prime }F^{\prime }H^{\prime }S^{-1}SS^{-1}HFWR$$

(110) $$=R(S^{-1}+W^{\prime }F^{\prime }H^{\prime }S^{-1}HFW)R$$

and with the optimal Kalman filter gain *W*, combined with (14), we get (107)-(110), as shown at the bottom of the next page.

Note that *E*[*s*(*k*)*s*(*k*)′] can be used in a manner similar to the algorithm in Section V to obtain the optimal Kalman filter gain *W*. More investigation is needed into this approach.

## VII. ESTIMATION OF *Q, P* AND $\bar{P}$

In this section, we discuss a method to estimate the process noise covariance *Q* and the state prediction (updated) covariance $\bar{P}$ (*P*). Unlike the case of a Wiener process and for a process with *H* = *I*, where both *Q* and $\bar{P}$ can be estimated separately and without iteration, as shown in Section IX-A3, *Q* and $\bar{P}$ (*P*) are coupled in the general case, requiring multiple iterations for the estimation to converge. The relationship between the steady-state state prediction covariance matrix $\bar{P}$ and the steady-state updated state covariance matrix *P* with the process noise covariance matrix *Q* is

(111) $$\bar{P}=FPF^{\prime }+\Gamma Q\Gamma^{\prime }$$

(112) $$=F{\left({\bar{P}}^{-1}+H^{\prime }R^{-1}H\right)}^{-1}F^{\prime }+\Gamma Q\Gamma^{\prime }$$

(113) $$=\bar{F}\bar{P}{\bar{F}}^{\prime }+FW\;RW^{\prime }F^{\prime }+\Gamma Q\Gamma^{\prime }$$

Similarly, the steady-state updated state covariance matrix can be written as

(114) $$P=\tilde{F}P{\tilde{F}}^{\prime }+WRW^{\prime }\;\;+(I_{n_{x}}-WH)\Gamma Q\Gamma^{\prime }(I_{n_{x}}-WH{)}^{\prime }$$

(115) $$={\left({\bar{P}}^{-1}+H^{\prime }R^{-1}H\right)}^{-1}$$

(116) $$={\left[(FPF^{\prime }+\Gamma Q\Gamma^{\prime }{)}^{-1}+H^{\prime }R^{-1}H\right]}^{-1}$$

where $\tilde{F}$ is defined as in (99) and (115) is derived utilizing (14) and the fact (from [2]) that

(117) $$P=(I_{n_{x}}-WH)\bar{P}$$

We also define $\tilde{P}$ as

(118) $$\tilde{P}≜FPF^{\prime }=\bar{F}\tilde{P}{\bar{F}}^{\prime }+FWRW^{\prime }F^{\prime }+\bar{F}\Gamma Q\Gamma^{\prime }{\bar{F}}^{\prime }$$

Given $\tilde{P}$ and *S*, or *P* and *S*, or $\bar{P}$ and *S*, we can compute Γ*Q*Γ′ in the following ways:

(119) $$Q1\;:\Gamma Q\Gamma^{\prime }=F^{-1}\tilde{P}(F^{-1}{)}^{\prime }+WSW^{\prime }-\tilde{P}$$

(120) $$Q2\;:\Gamma Q\Gamma^{\prime }=P+WSW^{\prime }-FPF^{\prime }$$

(121) $$Q3\;:\Gamma Q\Gamma^{\prime }=\bar{P}-F\bar{P}F^{\prime }+FWSW^{\prime }F^{\prime }$$

where **Q1** – **Q3** are derived from (6).

In this paper, we utilize the updated state covariance matrix to estimate *Q* and *P*, iteratively. Let *t* = 0, 1, … and *ℓ* = 0, 1, … denote the (two loop) iteration indices, and let us assume the initial estimate Γ*Q*^(0)^Γ′ = *WSW*′ (this is the Wiener process solution for the estimation of *Q*, as shown in Section IX). Let us initialize *P* by solving the Lyapunov equation (starting with *t* = 0 and *ℓ* = 0)

(122) $$P^{\left(0\right)}=\tilde{F}P^{\left(0\right)}{\tilde{F}}^{\prime }+WRW^{\prime }+(I_{nx}-WH)\;\Gamma Q^{\left(t\right)}\Gamma^{\prime }(I_{nx}-WH{)}^{\prime }$$

for *P*^(0)^. We compute *P*^(*ℓ*+1)^ utilizing (116) until the value converges, that is,

(123) $$P^{\left(\ell +1\right)}={\left[{\left(FP^{\left(\ell \right)}F^{\prime }+\Gamma Q^{\left(t\right)}\Gamma^{\prime }\right)}^{-1}+H^{\prime }R^{-1}H\right]}^{-1}$$

Given the converged *P*, let us denote *D*^(*t*+1)^ as

(124) $$D^{\left(t+1\right)}=P+WSW^{\prime }-FPF^{\prime }$$

Then, we can update *Q*^(*t*+1)^ from (120)

(125) $$Q^{\left(t+1\right)}=\Gamma^{†}D^{\left(t+1\right)}(\Gamma^{\prime }{)}^{†}$$

A mask matrix *A* can shape *Q* to enforce the structural constraints (e.g., diagonal covariance). The mask matrix comprises binary matrix elements with a 1 in the desired positions and 0, elsewhere, for example, as in an identity matrix. Then *Q* is structured by

(126) $$Q^{\left(t+1\right)}=A⊙Q^{\left(t+1\right)}$$

where ☉ is the Hadamard product. We subsequently set *ℓ* = 0 and recompute *P* using *Q*^(*t*+1)^ in (123), and this process repeats until the estimate of *Q* converges. For ill-conditioned systems, a tuning (regularization) parameter *λ_Q_* can be used in (125), that is

(127) $$Q^{\left(t+1\right)}=\Gamma^{†}\left[D^{\left(t+1\right)}+\lambda_{Q}I_{n_{x}}\right](\Gamma^{\prime }{)}^{†}$$

After the estimate of *Q* converges, we can estimate $\bar{P}$ using either (111), (112) or (113).

## VIII. ITERATIVE ALGORITHM TO ESTIMATE STEADY-STATE *W, S, P* $\left(\bar{P}\right)$, *Q* AND *R*

Given the methods to obtain estimates of *R* and *Q* in Sections VI and VII, we summarize our method into a six-step solution approach to obtain the optimal steady-state *W, S, P* $\left(\bar{P}\right)$, *Q*, and *R*.

### A. STEP 1

Start with iteration *r* = 0 and initialize with a *W*^(0)^ to stabilize the system as in [28]. We execute the Kalman filter for samples *k* = 1, 2, …, *N* as

(128) $${\hat{x}}^{\left(r\right)}(k+1|k)=F{\hat{x}}^{\left(r\right)}(k|k)$$

(129) $$v^{\left(r\right)}(k+1)=z(k+1)-H{\hat{x}}^{\left(r\right)}(k+1|k)$$

(130) $${\hat{x}}^{\left(r\right)}(k+1|k+1)={\hat{x}}^{\left(r\right)}(k+1|k)+W^{\left(r\right)}v^{\left(r\right)}(k+1)$$

(131) $$\mu^{\left(r\right)}(k+1)=z(k+1)-H{\hat{x}}^{\left(r\right)}(k+1|k+1)$$

### B. STEP 2

Compute *M* sample covariance matrices, as in (50).

### C. STEP 3

In this step, we check whether any of the termination conditions given below are met. If none of the termination conditions are met, we update the Kalman filter gain via the proposed method, detailed later in Section VIII-C2.

#### 1) TERMINATION CONDITIONS

There are five conditions that result in algorithm termination, subsequently yielding a Kalman filter gain *W* for *R, Q* and *P* estimates in later steps:

Condition 1: The converged Kalman filter gain is within a specified threshold *ζ*_*W*_.

Condition 2: The gradient of Kalman filter gain (60) is within a specified threshold *ζ*_Δ_.

Condition 3: The objective function value in (52) is within a specified threshold *ζ*_*J*_ from zero.

Condition 4: The objective function value stops improving, given a specified “patience” (number of epochs, detailed in Section VIII-C2) for the adaptive gradient method.

Condition 5: The maximum number of iterations is reached.

##### a: CONDITION 1

Let Δ*W* be the change in the Kalman filter gain from iteration *r* to *r* + 1, that is

(132) $$\Delta W=W^{\left(r+1\right)}-W^{\left(r\right)}$$

then

(133) $$\delta_{W}=||\Delta W_{\cdot }∕(W^{\left(r\right)}+\epsilon_{W})||$$

where ./ indicates element-wise division and ∥·∥ is a matrix norm (In this paper, the authors use the Euclidean norm) and *ϵ*_*W*_ is a very small value to protect against zeros in the denominator. When *δ*_*W*_ is less than a specified threshold *ζ*_*W*_, the Kalman filter gain is assumed to have converged and we terminate the algorithm; otherwise, we update the Kalman filter gain *W* for the next iteration.

##### b: CONDITION 2

We also examine the gradient of the Kalman filter gain ∇_*W*_*J* for convergence. We assume the Kalman filter gain to be converged when the Euclidean norm of ∇_*W*_*J* is less than a sufficiently small threshold *ζ*_Δ_, that is,

(134) $$\|\nabla_{W}J{\|}_{2}<\zeta_{\Delta }$$

##### c: CONDITION 3

Similar to *W*, we can compute the change in the objective function *J* from iteration *r* to *r* + 1. The Kalman filter gain is assumed to have converged when *J*^(*r*)^ is less than a specified threshold *ζ*_*J*_; otherwise, we update the Kalman filter gain for the next iteration.

##### d: CONDITION 4

The fourth termination condition is related to the step size for the proposed approximation method. We adapt the bold driver method in [3], [29], [53] and the method considers a “patience” parameter to indicate that the objective function value *J*^(*r*)^ has stopped improving (detailed in Section VIII-C2). The algorithm is terminated with the Kalman filter gain corresponding to minimum *J*^(*r*)^.

##### e: CONDITION 5

This condition is implemented to ensure that the algorithm terminates within a reasonable number of iterations, denoted by *n_L_*. Typically, the number of iterations required to reach the optimal Kalman filter gain *W* increases proportionally with *n_x_*.

#### 2) KALMAN FILTER GAIN UPDATE

When any of the above conditions are met, we terminate the algorithm. Otherwise, we update the Kalman filter gain *W* for the next iteration *r*+1 via the gradient direction in (60). Given the gradient direction, the Kalman filter gain at iteration *r*+1 is updated by

(135) $$W^{\left(r+1\right)}=W^{\left(r\right)}-\alpha^{\left(r\right)}\nabla_{W}J$$

where *α*^(*r*)^ is the step size for the proposed method. The step size is initialized as

(136) $$\alpha^{\left(0\right)}=\text{min}\left(c{\left(\frac{N}{N_{s}}\right)}^{\beta },c\right)$$

where *c* is a positive constant and is used to update the Kalman filter gain in the first iteration, *N_s_* is a hyperparameter on the number of observations, and *β* is a positive constant to adapt the initial step size to the number of observations. Note that (136) is selected to automatically tune the initial step size. When only a small subset of samples are observed, we want to use a smaller step size to prevent large steps that could result in unstable gains. If a line search is used instead, initialization is not necessary. Use of stochastic approximation type step sizes will enable one to extend the estimation method to on-line situations and the extended Kalman filter.

Subsequently, *α*^(*r*)^ is computed using the bold driver method in [3], [29], [53]. That is, after each iteration, we compare the *J*^(*r*)^ to its previous value, *J*^(*r*−1)^, and set

(137) α(r)={0.5α(r−1),ifJ(r)>J(r−1)max(1.1α(r−1),c¯),otherwise}

where $\bar{c}$ is the maximum step size defined as,

(138) $$\bar{c}=\text{min}\left({\left(\frac{N}{N_{s}}\right)}^{\beta },c_{\max}\right)$$

and *c*_max_ is a positive constant between 0 and 1.

Once we update the Kalman filter gain *W*, we go back to Step 1 by setting *r* = *r* + 1 and repeat the same process until any of the five termination conditions are met.

Note that each time *J*^(*r*)^ ≤ *J*^(*r*−1)^, we save the corresponding Kalman filter gain *W*^(*r*)^, and *J*^(*r*)^, and we halve the step size each time *J*^(*r*)^ > *J*^(*r*−1)^ in the hope of observing a decrease in *J*^(*r*)^. If the value of *J*^(*r*)^ has consecutively increased for a specified number of iterations (i.e., given a “patience” factor), we select the best Kalman filter gain *W* by

(139) $$W=\text{arg}\underset{r}{\text{min}}J^{\left(r\right)}$$

We then terminate the iteration and move onto Step 4 after repeating Steps 1 and 2 with the corresponding *W*. Note that adaptive stochastic gradient descent methods can be applied to compute the optimal Kalman filter gain *W* as in [21], [26], [40], [50], [54].

### D. STEP 4

Once we obtain the optimal steady-state Kalman filter gain *W* and the corresponding innovation covariance *S*, we can compute the unknown *R*, as in Section VI.

### E. STEP 5

Given the covariance matrix *R*, computed in Step 4, we can compute the covariance matrix *Q* and steady-state state prediction covariance matrix $\bar{P}$, as detailed in Section VII.

### F. STEP 6

We implement a successive approximation as follows: an outer-loop is used to reinitialize with the *R* and *Q* obtained from Step 5 and then reinvoke Steps 1-5. We keep track of the best *J*^(*r*)^ among the outer-loop iterations. The Kalman filter gain associated with the minimum *J*^(*r*)^ is selected to be the optimal Kalman filter gain. The algorithm terminates when the difference between the best *J*^(*r*)^ from each outer-loop is less than *ζ_J_* or the maximum number of outer-loop iterations is reached.

## IX. SPECIAL CASES: WIENER PROCESS AND *H* = *I*_*n*_*x*__ CASES

In this section, we consider two special cases below. The first case is when the state transition matrix *F* and the measurement matrix *H* are both identity matrices, *I*_*n*_*x*__ and *I*_*n*_*z*__, where *n_x_* = *n_z_*. This considerably simplifies our method to estimate *R* and *Q*. The second special case is when only the measurement matrix *H* is the identity matrix, while the state transition matrix *F* remains general. Note that we can extend either case to that of one assuming perfect measurements, that is, when *H* = *I*_*n*_*x*__, we have no measurement noise, and thus, *R* = 0.

### A. CASE 1: WIENER PROCESS

For a Wiener process, we have *F* = *I*_*n*_*x*__ and *H* = *I*_*n*_*x*__.

#### 1) KALMAN FILTER GAIN UPDATE FOR A WIENER PROCESS

To get the optimal Kalman filter gain, for *k* = 1, 2, …, *N*,

(140) $$\hat{x}(k|k-1)=\hat{x}(k-1|k-2)+W\;v(k-1)$$

(141) $$\hat{z}(k|k-1)=\hat{x}(k|k-1)$$

(142) $$z(k)=\hat{z}(k|k-1)+v(k)\;\;=\hat{x}(k|k-1)+v(k)$$

Define

(143) ξ(k)=z(k)−z(k−1)

(144) $$=\hat{x}(k|k-1)+v(k)-\hat{x}(k-1|k-2)\;\;-v(k-1)$$

(145) $$=v(k)+(W-I_{n_{x}})v(k-1)$$

Then, let us define *L*_0_ and *L*_1_ as

(146) $$L_{0}=E\;[\xi (k)\xi (k{)}^{\prime }]\;\;=S+(W-I_{n_{x}})S(W-I_{n_{x}}{)}^{\prime }$$

(147) $$L_{1}=E\;[\xi (k)\xi (k-1{)}^{\prime }]=(W-I_{n_{x}})S$$

Note that both *L*_0_ and *L*_1_ can be computed from samples. Additionally, we can obtain the optimal *W* from *L*_1_ as

(148) $$W=I_{n_{x}}+L_{1}S^{-1}$$

Substituting *W* in (148) into (146), we can write the relationship between *L*_0_ and *L*_1_ as

(149) $$L_{0}=S+L_{1}S^{-1}SS^{-1}L_{1}^{\prime }$$

(150) $$=S+L_{1}S^{-1}L_{1}^{\prime }$$

Note that (150) is in a form related to the discrete algebraic Riccati equation and has a positive definite solution [15].

#### 2) ESTIMATION OF *R* FOR A WIENER PROCESS

*Proposition 3:* For a Wiener process where both the state transition matrix *F* and the measurement matrix *H* are both the identity matrices, *I*_*n*_*x*__ and *I*_*n*_*z*__, respectively, where *n_x_* = *n_z_*, and given the optimal steady-state Kalman filter gain *W*, and the concomitant post-fit residual sequence *μ*(*k*) and innovation sequence *ν*(*k*), the covariance matrix *R* can be computed in the following ways:

(151) $$\mathrm{SR}1\;:R=(I_{n_{z}}-W)S$$

(152) $$\mathrm{SR}2\;:R=\frac{1}{2}\;\{E[\mu (k)v(k{)}^{\prime }+E[v(k)\mu (k{)}^{\prime }]\}$$

(153) $$\mathrm{SR}3\;:G=RS^{-1}R$$

(154) $$\mathrm{SR}4\;:R=\frac{1}{2}\;[G+S-WSW^{\prime }]$$

(155) $$\mathrm{SR}5\;:R=G-WSW^{\prime }+\frac{1}{2}(WS+S^{\prime }W^{\prime })$$

*Proof:* **SR1**-**SR4** are directly proven by substituting *H* = *I*_*n*_*z*__ into **R1–R4**. For **SR5**, we know from (8) that

(156) $$WS=\bar{P}$$

and we also know from (15) that,

(157) $$S=\bar{P}+R$$

Then,

(158) $$G=(I_{n_{x}}-W)S(I_{n_{x}}-W{)}^{\prime }$$

(159) $$=(I_{n_{x}}-W)S-(I_{n_{x}}-W)SW^{\prime }$$

(160) $$=S-WS-SW^{\prime }+WSW$$

(161) $$=R-SW^{\prime }+WSW^{\prime }$$

Then, we can compute *R* as

(162) $$R=G+WS-WSW^{\prime }$$

Symmetrizing (162),

(163) $$R=G-WSW^{\prime }+\frac{1}{2}(WS+S^{\prime }W^{\prime })$$

hence, **SR5** is proven.

#### 3) ESTIMATION OF $\bar{P}$ AND *Q* FOR A WIENER PROCESS

Unlike the general case, where multiple iterations are needed to estimate both *Q* and $\bar{P}$, in the case of a Wiener process, we can estimate them with no iteration.

*Proposition 4:* For a Wiener process, where the state transition matrix *F* and the measurement matrix *H* are both identity matrices, *I*_*n*_*x*__ and *I*_*n*_*z*__, respectively, and given the optimal steady-state Kalman filter gain *W*, and the corresponding innovation sequence *ν*(*k*), the steady-state state prediction covariance and the process noise covariance *Q* can be computed as:

(164) $$\bar{P}=WS$$

(165) $$Q=WSW^{\prime }$$

*Proof:* Given the relationship in (8) and knowing that, for a Wiener process *H* = *I*_*n*_*z*__, using (8), we have (164).

For a Wiener process, we can rewrite the Riccati equation (10) as

(166) $$\bar{P}=\bar{P}-\bar{P}(\bar{P}+R{)}^{-1}\bar{P}+Q$$

Using the relationship of (15) and (164) in (166) yields

(167) $$\bar{P}=\bar{P}-WSW^{\prime }+Q$$

Thus, for a Wiener process, *Q* can be estimated as

(168) $$Q=WSW^{\prime }$$

Hence, (165) is proven. Note that (165) is used as *Q*^(0)^ in the general case for iteratively computing *Q*. Also note that when *R* = 0 (i.e., perfect measurement case), we have,

(169) $$W=I_{n_{x}}$$

(170) P=0

(171) G=0

(172) $$Q=S=\bar{P}$$

### B. CASE 2: H = I_n_x__

In the second case, only *H* is the identity matrix, but *F* is not necessarily so.

#### 1) KALMAN FILTER GAIN UPDATE FOR THE *H* = *I*_*n*_*x*__ CASE

To get the optimal Kalman filter gain, for *k* = 1, 2, …, *N*,

(173) $$\hat{x}(k+1|k)=F\hat{x}(k|k-1)+FWv(k)$$

(174) $$z(k)=x(k)+w(k)\;\;=\hat{x}(k|k-1)+v(k)$$

Let *ξ*(*k*) be

(175) ξ(k)=z(k)−Fz(k−1)

Define

(176) $$\xi (k)=\hat{x}(k|k-1)+v(k)\;\;-F\hat{x}(k-1|k-2)-Fv(k-1)$$

(177) $$=v(k)-\bar{F}v(k-1)$$

where

(178) $$\bar{F}=F(I_{n_{x}}-W)$$

We can write *L*_0_ = *E*{*ξ*(*k*)*ξ*(*k*)′} as

(179) $$L_{0}=S+\bar{F}S{\bar{F}}^{\prime }$$

Similarly, *L*_1_ = *E*{*ξ*(*k*)*ξ*(*k* − 1)′} can be computed based on (177) as,

(180) $$L_{1}=-\bar{F}S$$

From the right hand side of (179), we can find *S* by solving

(181) $$S+L_{1}S^{-1}L_{1}^{\prime }=L_{0}$$

Upon calculating *S*, we can find the optimal Kalman filter gain *W* as

(182) $$W=I_{n_{z}}+F^{-1}L_{1}S^{-1}$$

and we can calculate *R* from *R*3, in (85). *G* can be obtained by running the filter given the optimal Kalman filter gain. Note that, we can also write *ξ*(*k*) as

(183) ξ(k)=x(k)−Fx(k−1)+w(k)−Fw(k−1)

(184) =Γv(k−1)+w(k)−Fw(k−1)

Then, *L*_0_ is

(185) $$L_{0}=\Gamma Q\Gamma^{\prime }+R+FRF^{\prime }$$

Equating (179) and (185), we can compute Γ*Q*Γ′ as

(186) $$\Gamma Q\Gamma^{\prime }=S+\bar{F}S{\bar{F}}^{\prime }-(R+FRF^{\prime })$$

(187) $$=S+FGF^{\prime }-(R+FRF^{\prime })$$

Equation (187) follows from

(188) $$G=(I_{n_{z}}-W)S(I_{n_{z}}-W{)}^{\prime }$$

Note that when *R* = 0 (i.e., perfect measurement case), we have *W*, *P* and *G* as in (169)-(171), respectively, and

(189) $$L_{0}=S=\Gamma Q\Gamma^{\prime }=\bar{P}$$

## X. NUMERICAL EXAMPLES

In this section, we illustrate our method on the following five cases:

1. A second-order kinematic system (a white noise acceleration or nearly constant velocity model) by varying the lags *M* in the correlation.
2. A system described in Neethling [39].
3. A five-state system from [33] and [4] with diagonal *Q* and *R*.
4. A detectable, but not completely observable, system from [42].
5. A three-state system from [42].

Each case is simulated with 100 Monte Carlo (MC) runs with an assumed “patience” of 5, *ζ_J_* = 10^−6^, *ζ_W_* = 10^−6^, *ζ*_Δ_ = 10^−6^, *c* = 0.01, *c*_max_ = 0.2, *β* = 2 and the maximum outer-loop iteration limit is set to 20. Case 5 is simulated with 200 MC runs to be compatible with the results in [42].

For each test case, we examine the condition number of the system’s observability and controllability matrices, as well as matrix I. The condition number of matrix *A* is computed as

(190) $$\kappa (A)=\|A\|\|A^{†}\|$$

where *A*^†^ is the pseudoinverse of *A* and ∥·∥ is a Euclidean norm. The rank of matrix I is also examined for each test case. For each test case result, we compute the 95% probability interval (PI) via the highest probability interval method^7^ and denote by $\underline{r}$ and $\bar{r}$ the corresponding lower and upper limits, respectively. We also provide the mean and the root mean squared error (RMSE) of each distribution. The averaged normalized innovation squared (NIS) is also provided to measure the consistency of the Kalman filter,

(191) $$\bar{\epsilon }(k)=\frac{1}{n_{MC}}\sum_{i=1}^{n_{MC}}v(k{)}^{\prime }S^{-1}v(k)$$

where *n_MC_* is the number of MC runs. The elements of each matrix *A* are denoted as *a_ij_*, representing the element in the *i^th^* row and the *j^th^* column of *A*.

### A. CASE 1

We simulated a second-order kinematic system described by

(192) $$x(k)=\left[\begin{array}{cc} 1 & T\\ 0 & 1 \end{array}\right]x(k-1)+\left[\begin{array}{c} \frac{1}{2}T^{2}\\ T \end{array}\right]v(k-1)$$

(193) z(k)=[10]x(k)+w(k)

with sampling period *T* = 0.1, where

(194) $$E[v(k)v(j{)}^{\prime }]=0.0025\delta_{kj}$$

(195) $$E[w(k)w(j{)}^{\prime }]=0.01\delta_{kj}$$

where *δ_kj_* is the Kronecker delta function. The mean of the process and the measurement noises are assumed to be zero and the corresponding variances are given in (194) and (195), respectively. Note that the system has the condition number of 20.1 for its observability matrix and 20.2 for its controllability matrix. The noise covariance identifiability matrix I, given the initial Kalman filter gain in (197), is

(196) $$I=\left[\begin{array}{cc} 5\cdot 10^{-5} & 6\\ 2.5\cdot 10^{-5} & -4\\ 0 & 1 \end{array}\right]$$

which has a rank of 2, and we have 2 unknown variables to estimate, implying that *Q* and *R* are identifiable. The condition number for I is 1.5·10^5^. The least squares problem using the minimal polynomial approach is ill-conditioned.

#### 1) VARYING THE NUMBER OF LAGS IN THE CORRELATIONS

**FIGURE 1.**
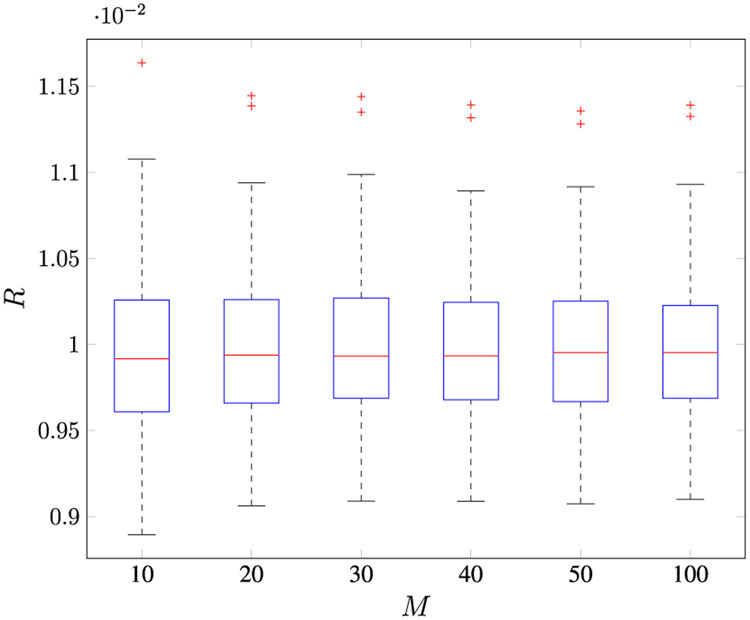
100 Monte Carlo runs for the Kalman filter *R* estimation using method R3 with various *M*.

**FIGURE 2.**
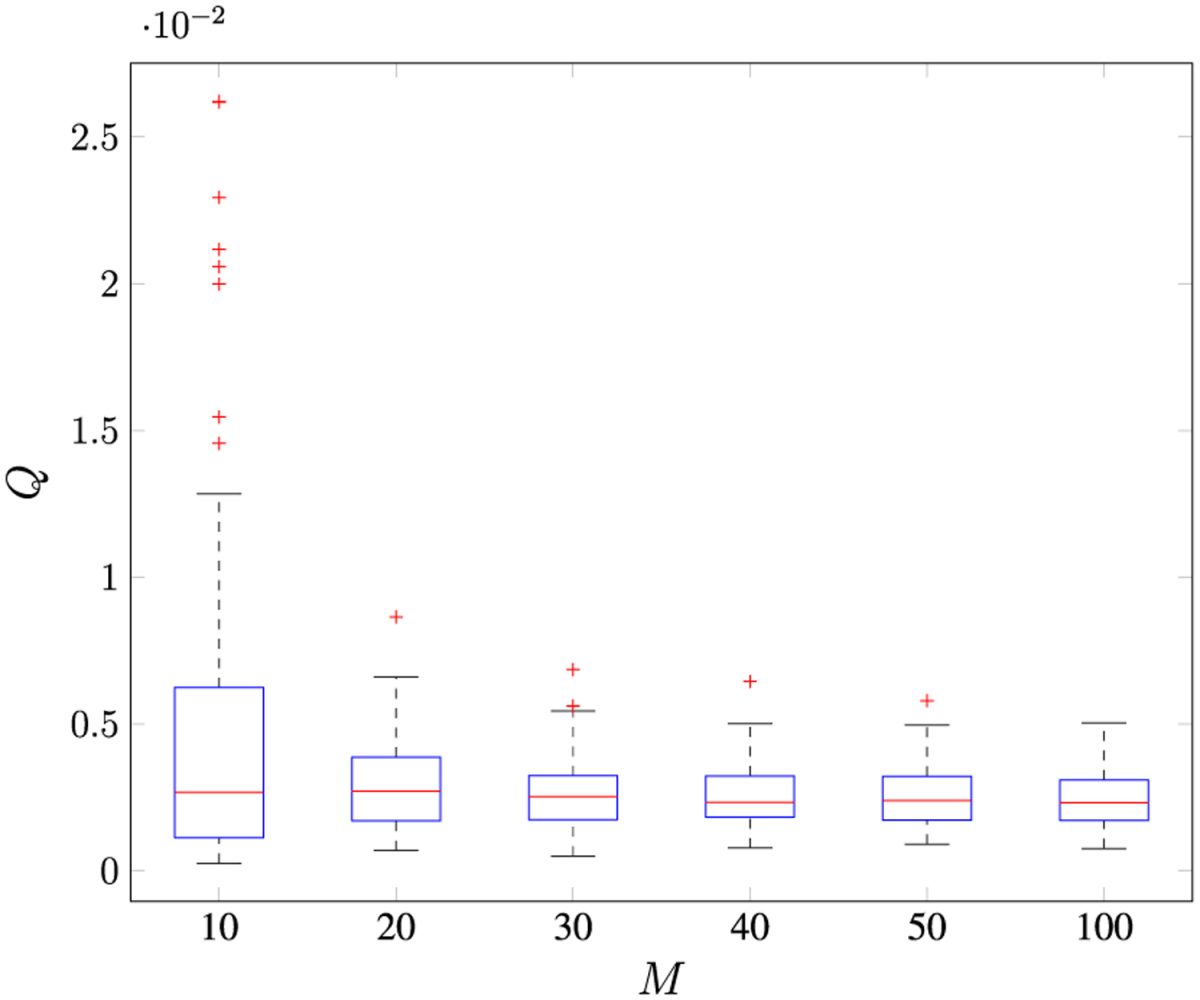
100 Monte Carlo runs for the Kalman filter *Q* estimation with various *M*.

We performed 100 MC runs, where each run contained *N* = 1000 sample observations. We set *n_L_* = 100, *N_s_* = 1000, and vary the lags, *M* = 10, 20, 30, 40, 50, 100, with an initial Kalman Filter gain

(197) $$W^{\left(0\right)}=\left[\begin{array}{c} 0.1319\\ 0.0932 \end{array}\right],$$

obtained by solving the Riccati equation with *Q*^(0)^ = 0.1 and *R*^(0)^ = 0.1. Figs.1 and 2 show the box plots of the estimated *R* using **R3**^8^ and *Q* of 100 MC runs, respectively, with varying *M*.

The bottom and top of each “box” are the first (denoted $Q_{1}$) and third (denoted $Q_{3}$) quartiles of the estimate, respectively. The line in the middle of each box is the median estimate. The distances between the tops and bottoms are the interquartile ranges $\left(\text{IQR}=Q_{3}-Q_{1}\right)$. The whiskers are lines extending above and below each box and are drawn from each end of the interquartile ranges to the upper $\left(Q_{3}+1.5\text{IQR}\right)$ and lower $\left(Q_{1}-1.5\text{IQR}\right)$ adjacent values. Estimates beyond the whisker length are marked as outliers (indicated by the “+” symbols). The accuracies of the estimates of both *R* and *Q* increase with an increase in *M*. Table 2 shows the mean value of the estimates of both *R* and *Q*. The smallest error of the median of the estimates of *R* and the variability of the estimates of *Q* are obtained with *M* ≥ 100.

#### 2) ESTIMATION OF *W* AND $\bar{P}$

**FIGURE 3.**
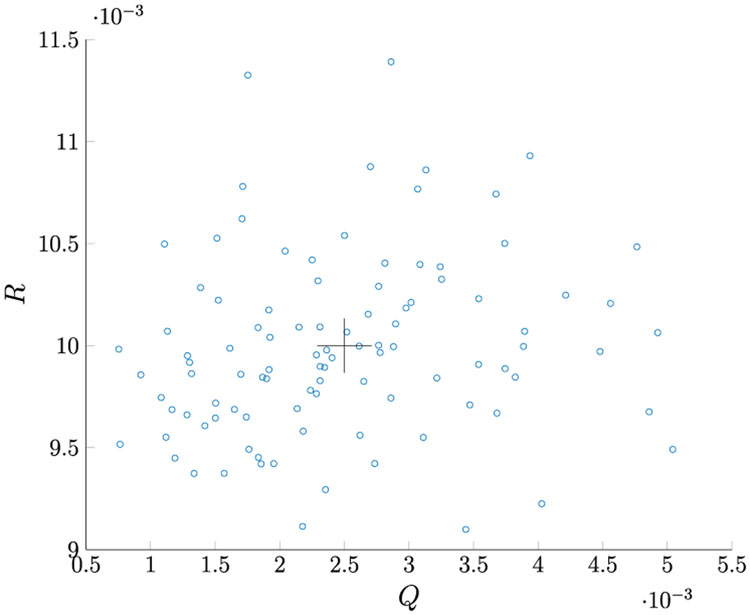
*Q* and *R* estimation for Case 1.

**FIGURE 4.**
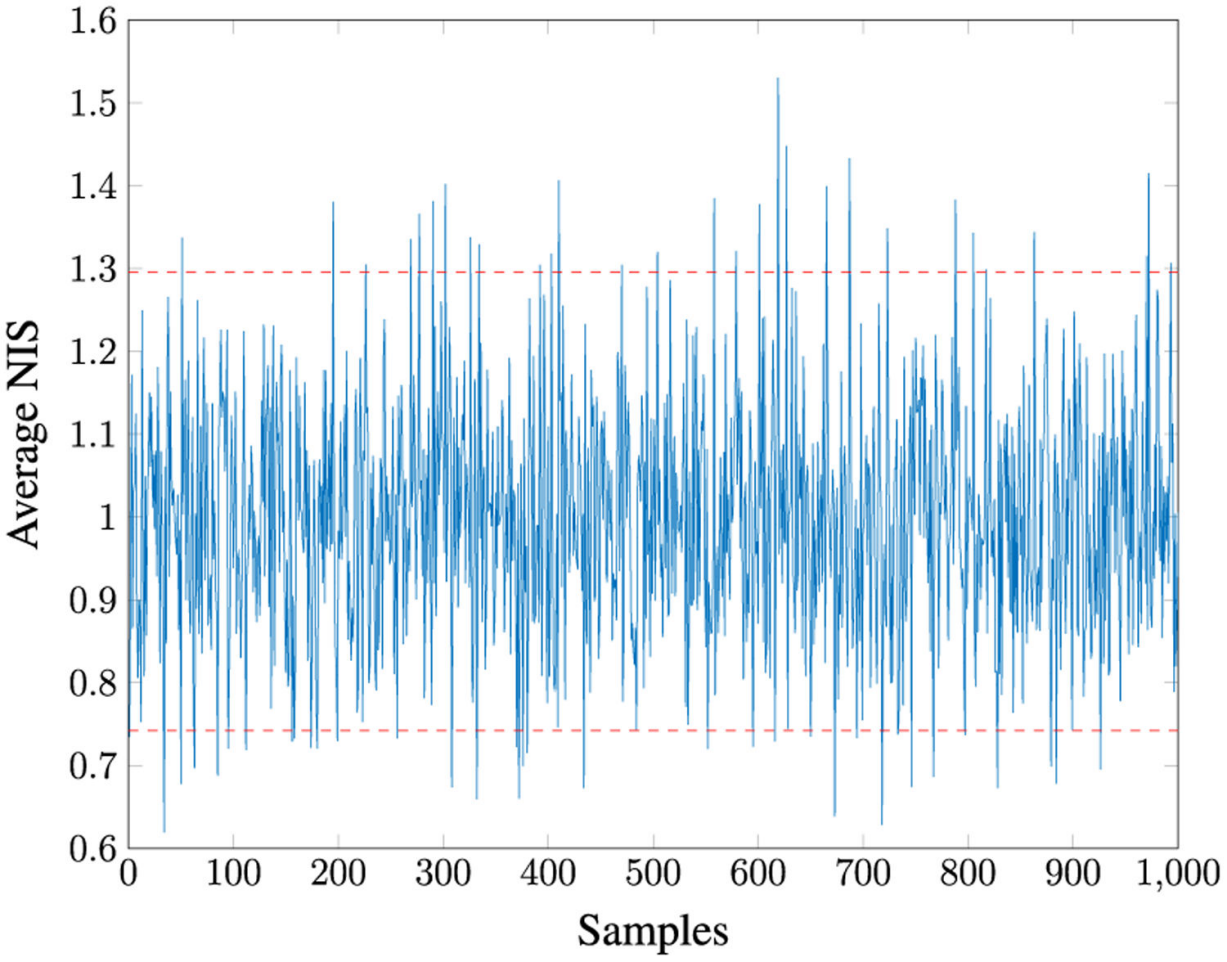
Averaged NIS for Case 1.

Given *M* = 100, for 100 MC runs with the initial Kalman Filter gain as in (197), we found that **R1–R5** estimate the same *R* values. The true values of *R* all lie within the 95% PI associated with the distribution of estimates. Fig.3 shows the *Q* versus *R* plot of each estimate. The true values are marked by “+” symbols. The reason the estimated *Q* varies so much is that its value is very small compared to the measurement noise. Fig.4 shows the averaged NIS and its 95% probability region, which proves that the filter is consistent.

### B. CASE 2

We simulated the system described in Neethling [39],

(198) $$x(k)=\left[\begin{array}{cc} 0.8 & 1\\ -0.4 & 0 \end{array}\right]x(k-1)+\left[\begin{array}{c} 1\\ 0.5 \end{array}\right]v(k-1)$$

(199) z(k)=[10]x(k)+w(k)

where

(200) $$E[v(k)v(j{)}^{\prime }]=\delta_{kj}$$

(201) $$E[w(k)w(j{)}^{\prime }]=\delta_{kj}$$

The system’s condition numbers for its observability and controllability matrices are 2.18 and 2.56, respectively. Here, I, given the initial Kalman filter gain, is

(202) $$I=\left[\begin{array}{cc} 1.25 & 1.8\\ 0.5 & -1.12\\ 0 & 0.4 \end{array}\right]$$

and the rank is 2. The number of unknown variables is 2, therefore, the system noise variances are estimable. The condition number of I is 2.3 and indeed the minimal polynomial approach works well for this problem. We simulated 100 Monte Carlo runs with *N* = 1000, *n_L_* = 100, *N_s_* = 1000, and an initial suboptimal Kalman filter gain

(203) $$W^{\left(0\right)}=\left[\begin{array}{c} 0.9\\ 0.5 \end{array}\right]$$

**FIGURE 5.**
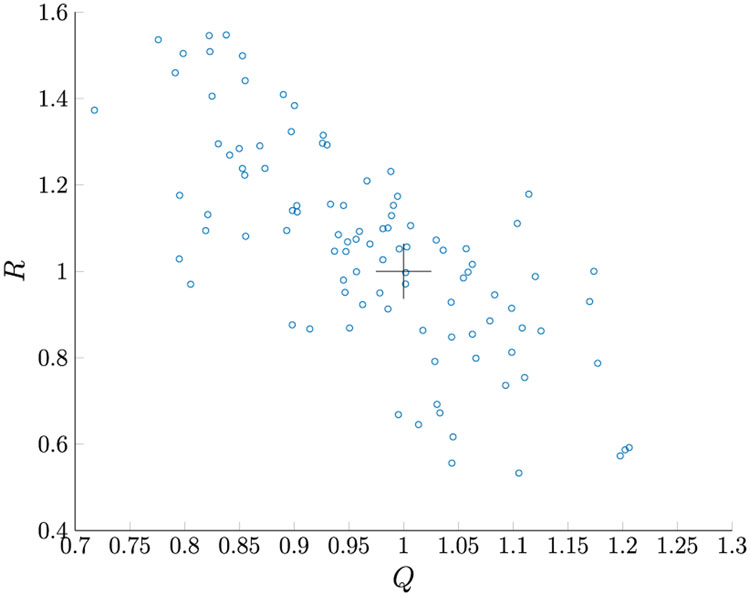
*Q* and *R* estimation for Case 2.

**FIGURE 6.**
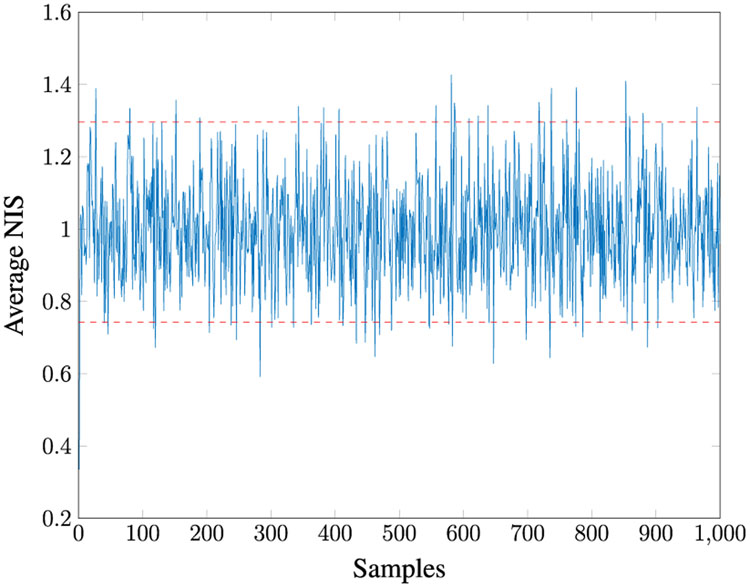
Averaged NIS for Case 2.

Table 4 shows the estimated noise variances. Similar to the Case 1 result, the mean values of each of the estimated parameters are very close to their corresponding true values. As seen in Table 4, the true values lie within the 95% PI associated with the distribution of estimates for each variable *Q*, *R*, *W* and *P_ii_*. Fig.5 shows the *Q* and *R* estimates for each MC run. As shown in Fig.6, the Kalman filter is considered consistent.

### C. CASE 3

In Case 3, we test on the example in [33]. The system matrices are assumed to be as follows.

(204) $$F=\left[\begin{array}{ccccc} 0.75 & -\;1.74 & -\;0.3 & 0 & -\;0.15\\ 0.09 & 0.91 & -\;0.0015 & 0 & -\;0.008\\ 0 & 0 & 0.95 & 0 & 0\\ 0 & 0 & 0 & 0.55 & 0\\ 0 & 0 & 0 & 0 & 0.905 \end{array}\right]$$

(205) $$\Gamma =\left[\begin{array}{ccc} 0 & 0 & 0\\ 0 & 0 & 0\\ 24.64 & 0 & 0\\ 0 & 0.835 & 0\\ 0 & 0 & 1.83 \end{array}\right]$$

(206) $$H=\left[\begin{array}{ccccc} 1 & 0 & 0 & 0 & 1\\ 0 & 1 & 0 & 1 & 0 \end{array}\right]$$

(207) $$Q=\left[\begin{array}{ccc} 1 & 0 & 0\\ 0 & 1 & 0\\ 0 & 0 & 1 \end{array}\right]\;\;R=\left[\begin{array}{cc} 1 & 0\\ 0 & 1 \end{array}\right]$$

The condition number for the observability matrix is 42.6, and the condition number for the controllability matrix is 54.6. The system has a rank (I) equal to 5 (utilizing the constraint that both *R* and *Q* are diagonal), with a total of 5 unknowns. Hence, the *Q* and *R* parameters are identifiable. The condition number of the noise covariance identifiability matrix I is 808. The initial Kalman filter gain is obtained by solving the Riccati equation with

(208) $$Q^{\left(0\right)}=\left[\begin{array}{ccc} 0.25 & 0 & 0\\ 0 & 0.5 & 0\\ 0 & 0 & 0.75 \end{array}\right]$$

(209) $$R^{\left(0\right)}=\left[\begin{array}{cc} 0.4 & 0\\ 0 & 0.6 \end{array}\right]$$

#### 1) MINIMUM NUMBER OF OBSERVATION SAMPLES NEEDED FOR MEHRA’s AND Bélanger’s METHODS TO CONVERGE

**FIGURE 7.**
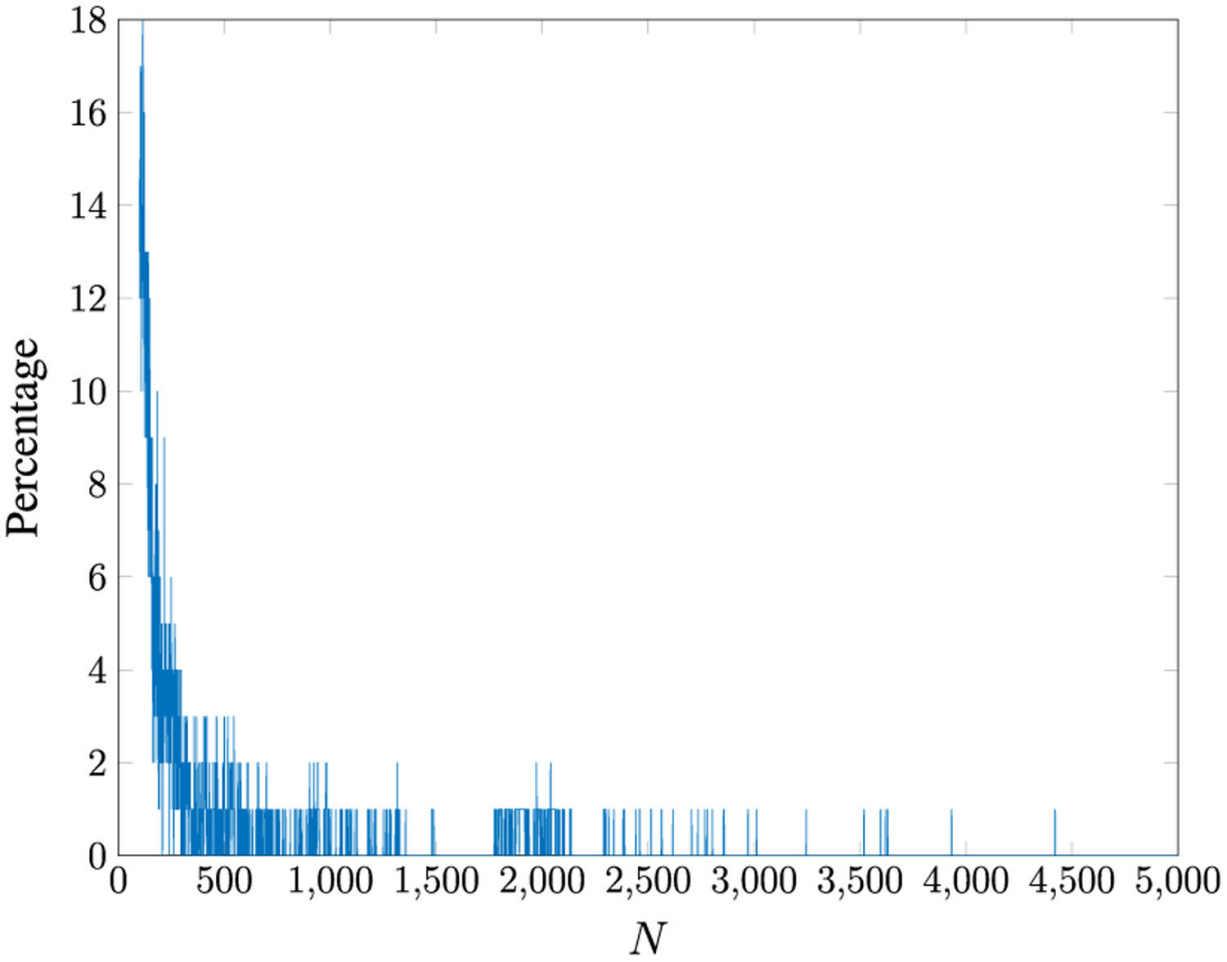
Percentage of unstable Kalman filter gains obtained from [33] for varying the total number of observed samples (*M* = 40).

**FIGURE 8.**
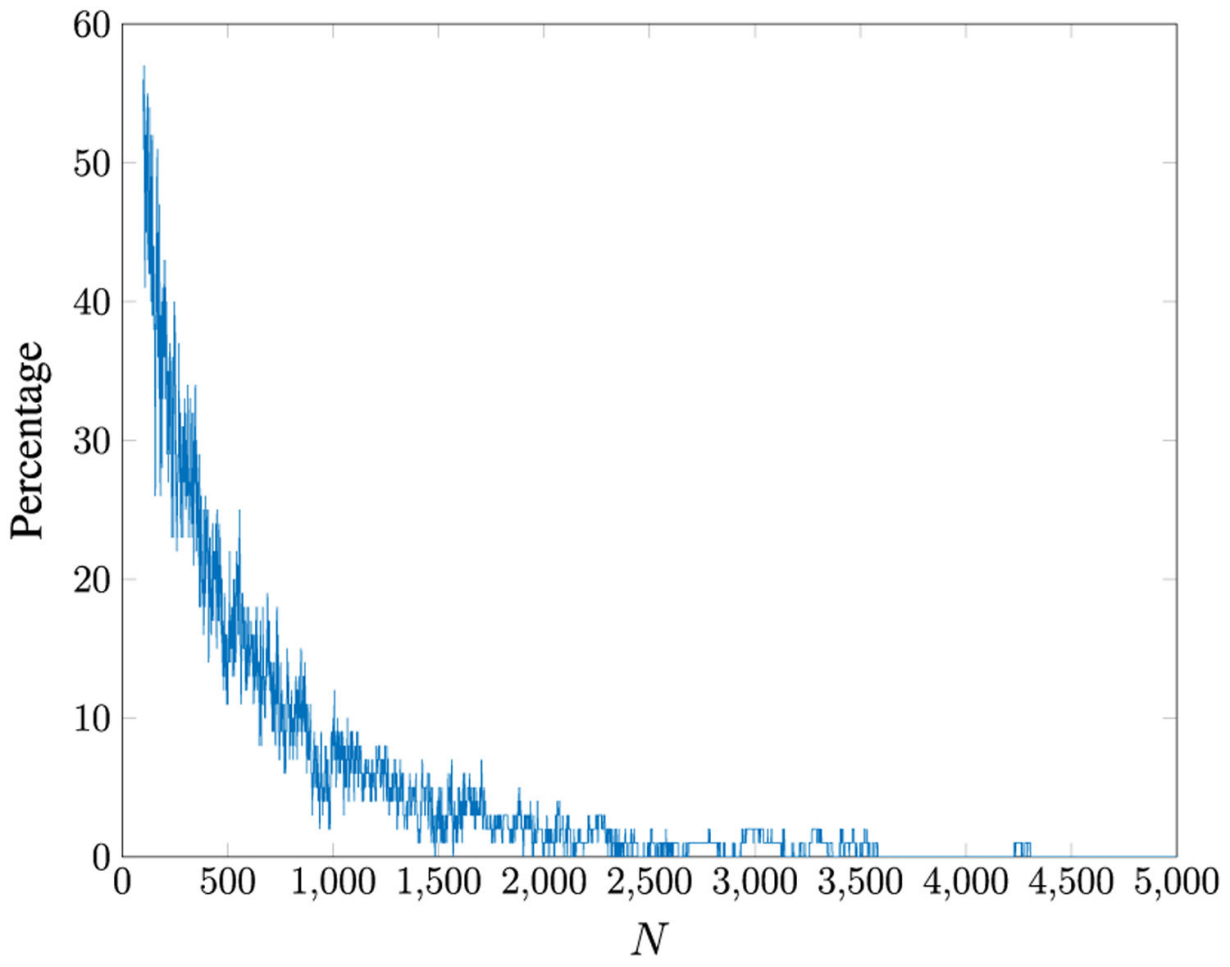
Percentage of unstable Kalman filter gains obtained from [4] for varying the total number of observed samples (*M* = 5).

Both Mehra’s [33] and Bélanger’s [4] methods to update the Kalman filter gain *W* can be unstable unless a large number of data samples are observed. This is due to the fact that the time average converges slowly to the ensemble average. We conducted 100 MC simulations with 10,000 data samples in each run given the five-state system described in (204)-(207). We then varied the number of observed samples from 100 to 10,000 and updated the Kalman filter gain using both the Mehra [33] and Bélanger [4] methods. We measure the percentage of unstable Kalman filter gains by checking if any of the eigenvalues of $\bar{F}$ are outside of the unit circle for each run over the 100 MC runs. The results are shown in Figs.7 and 8. We only display up to 5,000 samples for both methods because each approach terminated with a stable gain when the total observation samples exceeded 5,000. The minimum number of samples required to obtain a stable gain from these methods were about 4,500. Our proposed method always results in a stable Kalman filter gain; hence, it is not included in the comparison of methods.

#### 2) COMPARISON OF PROPOSED, MEHRA’S, AND BELANGER’S GAIN UPDATE METHODS

Given the 100 MC simulations with 10,000 observation samples generated in X-C1 and setting *n_L_* = 500, *N_s_* = 10000, Table 5 shows the estimation of the Kalman filter gain *W* over 100 Monte Carlo runs, given three different gain update methods: the proposed method with *M* = 40, Mehra’s method with *M* = 40 [33] and Bélanger’s method with *M* = 5 [4].

In Table 5, we see that all methods have the true values staying within its 95% PI; however, our proposed method is able to obtain the Kalman filter gain closest to the optimal Kalman filter gain and the RMSE are, on average, 8 and 4 times smaller compared to Mehra’s and Bélanger’s, respectively. The very small gains *W*_21_ and *W*_41_ are (similarly to the small *Q* from Case 1) very hard to estimate — they are essentially buried in noise.

We test and compare the proposed method with that of Mehra’s and Bélanger’s for the estimation of *R*, *Q* and *P* using the methodology described in Sections VI-B and VII, combined with the converged Kalman filter gain from Table 5. The results are shown in Table 6 and Mehra’s method results in the true value of *P*_33_ staying outside of the 95% PI. In comparison to Bélanger’s method, the proposed method is vastly more accurate with lower RMSE (2 to 9 times smaller) for all *R*, *Q*, and $\bar{P}$, while Mehra’s method obtained a result that is less accurate than Bélanger’s method, as expected from the Kalman filter gain results. The reason *r*_1_ is so difficult to estimate is that *S*_1_ is dominated by the state uncertainty (*S*_1_ = 65, *r*_1_ = 1), i.e. the measurement noise is “buried” in a much larger innovation. In the case of *r*_2_ = 1, one has *S*_2_ = 2.45, i.e., *r*_2_ is “visible” in the innovations.

#### 3) VARYING THE NUMBER OF SAMPLES OBSERVED

**FIGURE 9.**
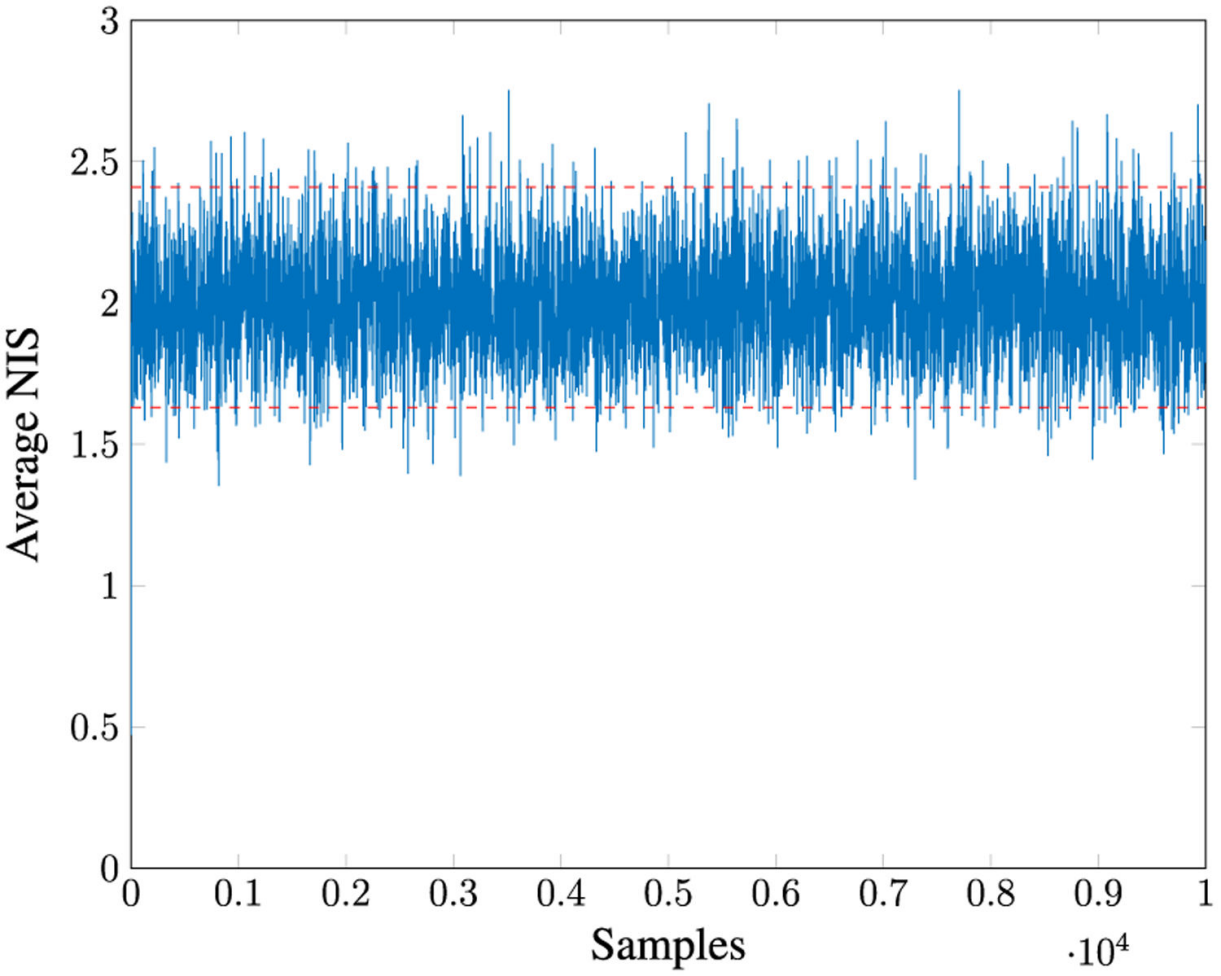
Averaged NIS for Case 3.

In this section, we vary the number of samples observed, *N* = 1000, 2500, 5000, 10000 using our six-step approach. The results are detailed in Tables 7 and 8. As expected, the accuracy increases with an increase in *N*. The estimation is greatly degraded for *N* < 5000. Fig.9 illustrates that the Kalman filter is consistent.

### D. CASE 4

For case 4, we simulate the unobservable (but detectable) system in [42],

(210) $$x(k)=\left[\begin{array}{cc} 0.1 & 0\\ 0 & 0.2 \end{array}\right]x(k-1)+\left[\begin{array}{c} 1\\ 2 \end{array}\right]v(k-1)$$

(211) z(k)=[10]x(k)+w(k)

with

(212) $$E[v(k)v(j{)}^{\prime }]=\delta_{kj}$$

(213) $$E[w(k)w(j{)}^{\prime }]=\delta_{kj}$$

With the initial Kalman filter gain, the system has

(214) $$I=\left[\begin{array}{cc} 1.04 & 1.09\\ -0.20 & -0.31\\ 0 & 0.02 \end{array}\right]$$

**FIGURE 10.**
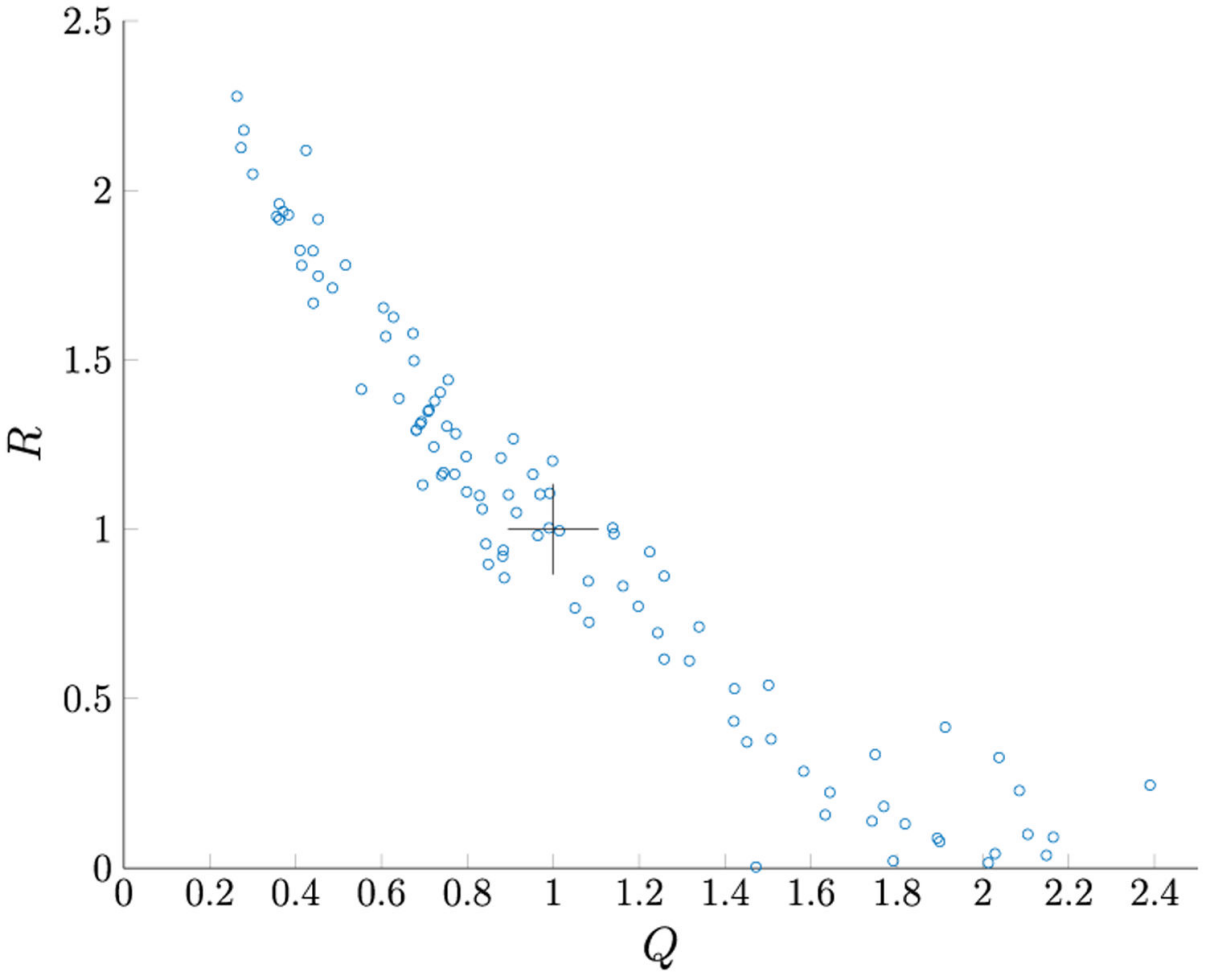
*Q* and *R* estimation for Case 4.

**FIGURE 11.**
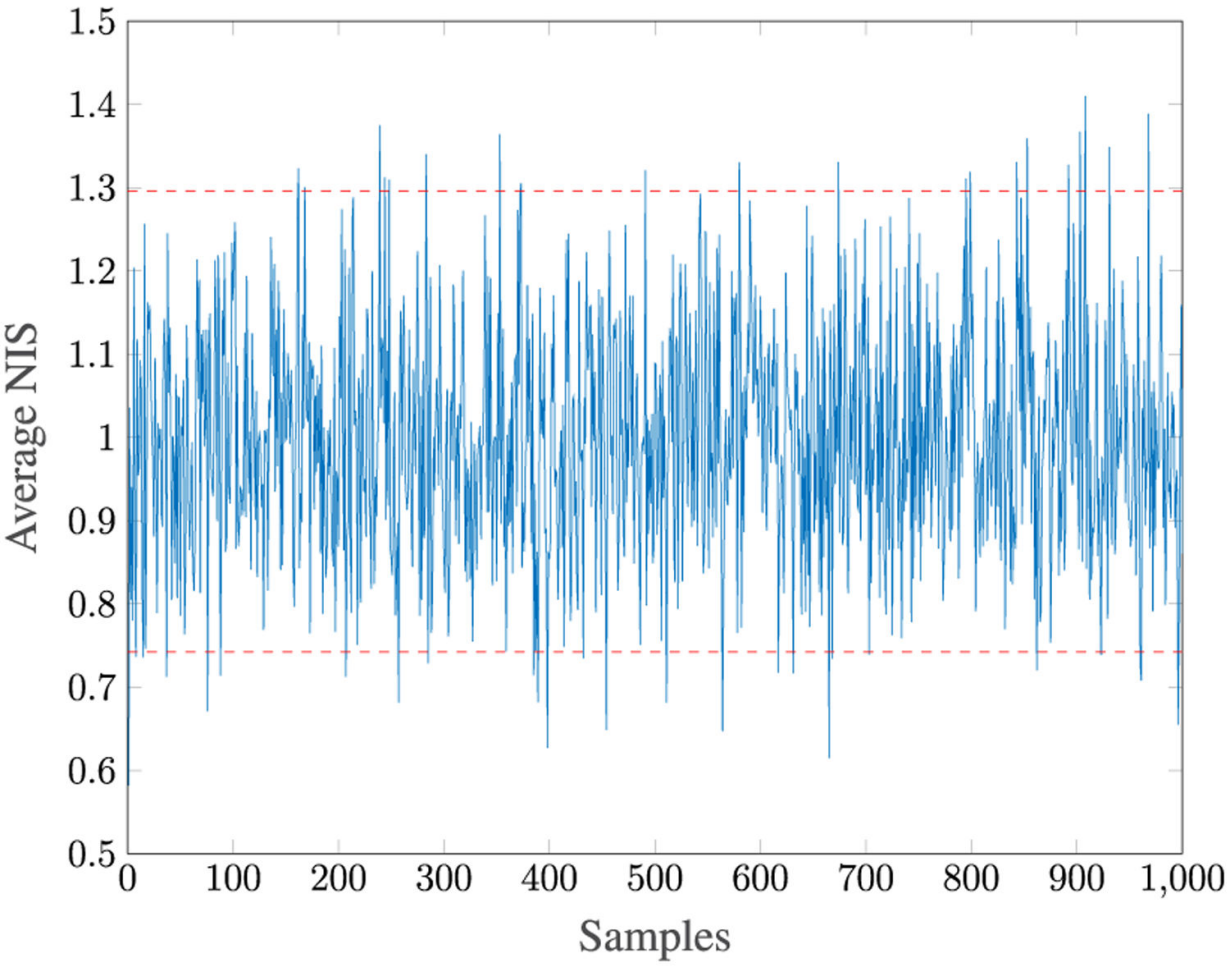
Averaged NIS for Case 4.

The rank of I is 2 and we have a total of 2 unknown variables. The condition number for I is 23.4. We simulated 100 MC runs with observed samples *N* = 1000 in each run. We set *n_L_* = 100, *N_s_* = 1000, and *λ_Q_* = 0.1. Table 9 shows the estimated parameters with the initial Kalman filter gain obtained by solving the Riccati equation with *R*^(0)^ = 0.2, and *Q*^(0)^ = 0.4. Note that the system is *not* fully observable, i.e., the condition number for the observability matrix is infinity, while that for the controllability matrix is 25.8. In Table 9, the true values lie within the 95% PI associated with each distribution. Fig.10 shows a wide variation of *Q* and *R* estimates; however, the NIS in Fig.11 shows that the Kalman filter is consistent.

### E. CASE 5

In Case 5, we simulate the system from [42],

(215) $$x(k)=\left[\begin{array}{ccc} 0.1 & 0 & 0.1\\ 0 & 0.2 & 0\\ 0 & 0 & 0.3 \end{array}\right]x(k-1)+\left[\begin{array}{c} 1\\ 2\\ 3 \end{array}\right]v(k-1)$$

(216) z(k)=[0.10.20]x(k)+w(k)

with

(217) $$E[v(k)v(j{)}^{\prime }]=0.5\delta_{kj}$$

(218) $$E[w(k)w(j{)}^{\prime }]=0.1\delta_{kj}$$

The condition number for observability and controllability matrices are 362 and 561, respectively; hence it is an ill-conditioned case. With the initial Kalman filter gain, the noise covariance identifiability matrix I is

(219) $$I=\left[\begin{array}{cc} 0.28 & 1.37\\ -0.09 & -0.67\\ 0.006 & 0.11\\ 0 & -0.006 \end{array}\right]$$

**FIGURE 12.**
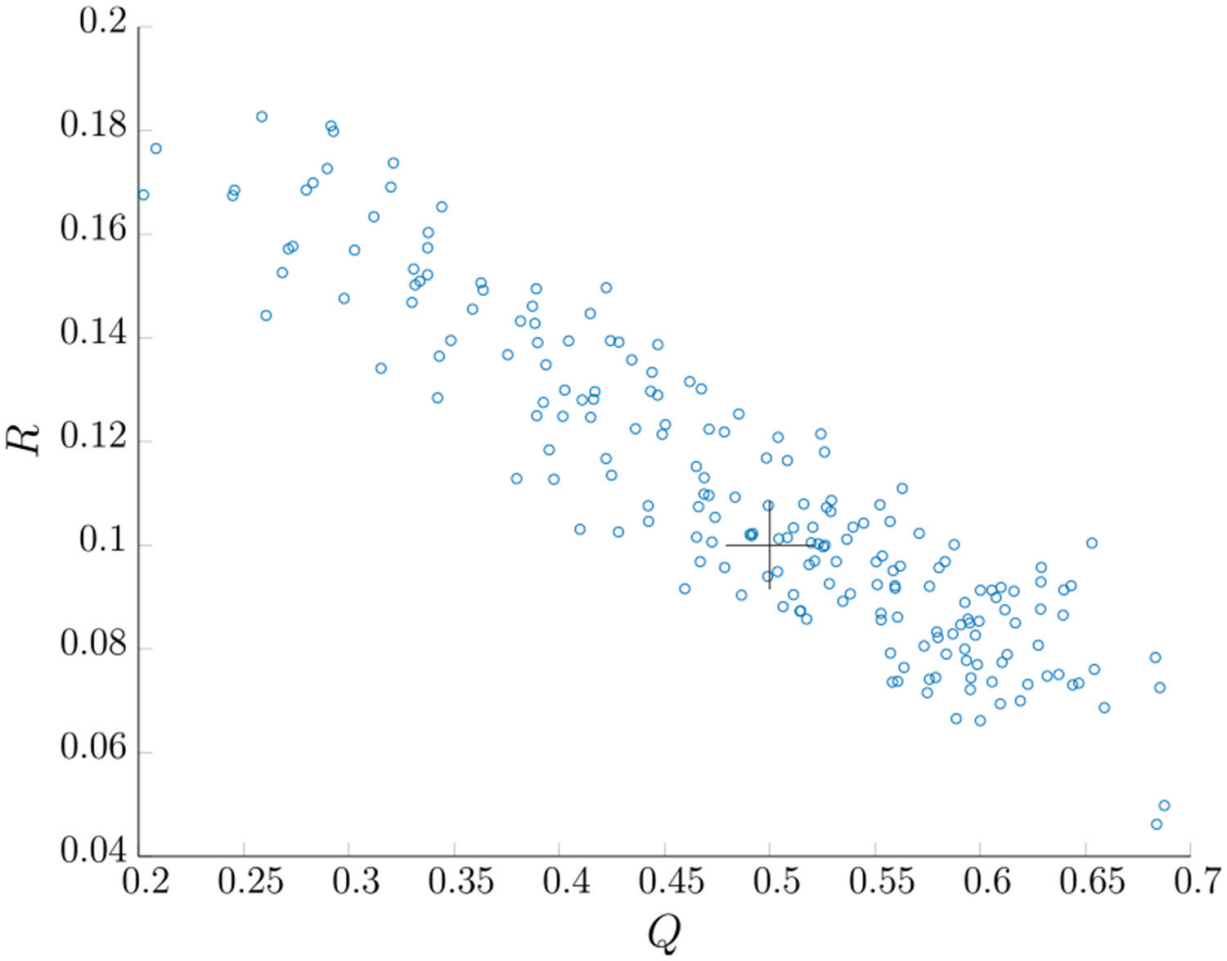
*Q* and *R* estimation for Case 5.

**FIGURE 13.**
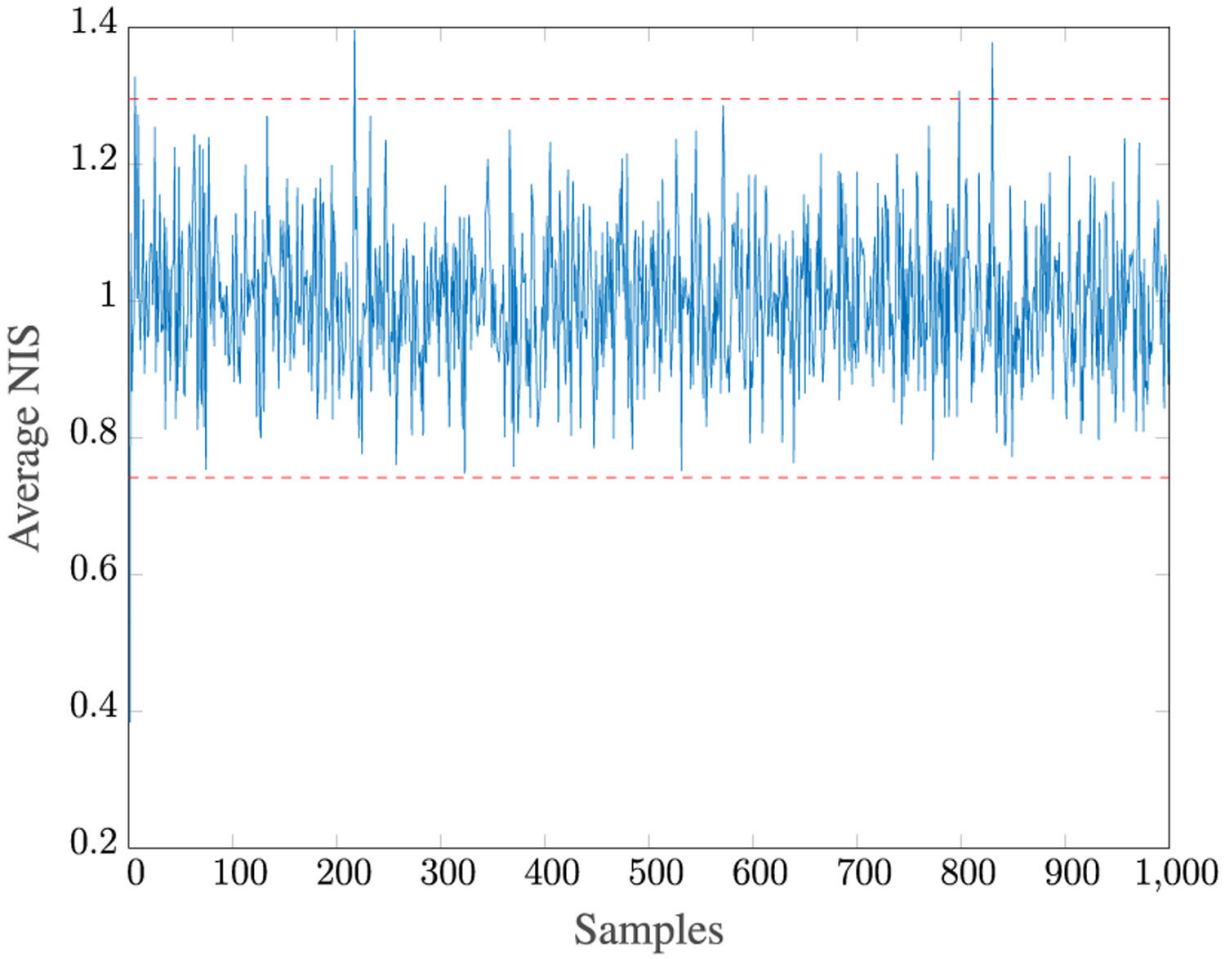
Averaged NIS for Case 5.

The rank of I is 2 and we have a total of 2 unknown variables indicating that both *Q* and *R* are identifiable (albeit due to the high condition number, not very well relative to the other systems tested). The condition number for I is 36.4. We simulated 200 MC runs with *N* = 1000 observed samples for each run. We set *M* = 15 to be consistent with the setup in [42]. We also set the maximum number of iterations *n_L_* = 100, *N_s_* = 1000, and the regularization term from (127) is *λ_Q_* = 0.3. Table 10 shows the estimated parameters with the initial Kalman filter gain obtained by solving the Riccati equation with *R*^(0)^ = 0.1, and *Q*^(0)^ = 0.5. The results are detailed in Table 10, where the true value stays within the 95% PI. Fig.12 shows the scatter plot for the estimates of *R* and *Q* of each MC run. The plot is similar to the estimates in [42]. However, the upper bound on *Q* is less than that of [42] (about 0.2), which does not provide the detailed results presented in Table 10. Fig.13 shows that the Kalman filter is consistent.

## XI. CONCLUSION AND FUTURE WORK

In this paper we derived necessary and sufficient conditions for the identification of the process and measurement noise covariances for a Gauss-Markov system. We also provide a novel six-step successive approximation method, coupled with an adaptive gradient method, to estimate the steady-state Kalman filter gain *W*, unknown noise covariance matrices *R*, and *Q*, as well as the state prediction (or updated) covariance matrix $\bar{P}$ (or *P*) when *Q* and *R* are identifiable. Moreover, we developed a novel iterative approach to obtain positive definite *Q*, *R* and $\bar{P}$, while ensuring that the structural assumptions on *Q* and *R* are enforced (e.g., diagonality of *Q* and *R*, if appropriate, symmetry and positive definiteness). We provided several approaches to estimate the unknown noise covariance *R* via *post-fit residuals*. We examined previous methods from the literature and heretofore undiscussed assumptions of these methods that result in largely inaccurate or unstable estimates of the unknown parameters. The proposed method significantly outperformed the previous ones, given the same system assumptions.

We validated the proposed method on five different test cases and were able to obtain parameter estimates where the truth stays within the 95% probability interval based on the estimates.

In the future, we plan to pursue a number of research avenues, including 1) estimating *Q* and *R* using one-step lag smoothed residuals; 2) exploring vector moving average estimation algorithms using the minimal polynomial approach and/or truncating the effects of state; 3) replacing the batch innovation covariance estimates by their individual or mini-batch estimates, as is done in machine learning, to enable real-time estimation; 4) investigating accelerated gradient methods (e.g., Adam [26], AdaGrad [13], RMSProp [50], conjugate gradient, memoryless quasi-Newton, and trust region methods [5]); 5) automatic model selection from a library of models; and 6) extension to nonlinear dynamic models.

## Figures and Tables

**TABLE 1.** Summary of notation.

*F*	state transition matrix of the system
*H*	measurement matrix
*J*	objective function value
*M*	number of lags in sample covariance matrix computation
*n_x_*	dimension of the vector *x*
*N*	number of observed samples
*P*	steady-state updated state covariance matrix
$\bar{P}$	steady-state state prediction covariance matrix
*v*	zero-mean white Gaussian noises with covariance matrices *Q*
*w*	zero-mean white Gaussian noises with covariance matrices *R*
*W*	Kalman filter gain
*x*	state vector
$\hat{x}$	estimate of *x*
$\tilde{x}$	error corresponding to the estimate of *x*
*z*	measurement vector
*α*	stepsize
Γ	noise gain matrix
$\bar{\epsilon }$	averaged NIS
*λ*	regularization parameter
*μ*	post-fit residual sequence with covariance matrix *G*
*ν*	innovation sequence with covariance matrix *S*
I	noise covariance identifiability matrix
Cov	covariance
*δ_kj_*	Kronecker delta function
diag(·)	diagonal of a matrix
*E*[·]	expectation
tr(·)	trace (of a matrix)
vec(·)	linear transformation to convert a matrix into a column vector
∣·∣	determinant (of a matrix)
∥·∥	norm of a matrix
$\{\nu (k){\}}_{k\underset{\;≜}{=1}}^{N}$	the sequence *ν*(*k*), *k* = 1, …, *N*
≜	equal by definition
∇_*W*_	gradient with respect to (the matrix) *W*
RMSE	root mean square error
NIS	normalized innovation squared

**TABLE 2.** Monte carlo simulation for Case 1 varying the number of lags *M* (Method R3).

	*M*					
	10	20	30	40	50	100
*R*	0.0100	0.0100	0.0100	0.0100	0.0100	0.0100
*Q*	0.0048	0.0030	0.0027	0.0026	0.0025	0.0025

**TABLE 3.** Monte Carlo Simulation for Case 1 with *M* = 100 and PI= 2*σ* (100 Runs).

	*R*					*Q*
	R1	R2	R3	R4	R5	
Truth	0.01	0.01	0.01	0.01	0.01	0.0025
$\underline{r}$	0.0092	0.0092	0.0092	0.0092	0.0092	6.49 · 10^−4^
Mean	0.0100	0.0100	0.0100	0.0100	0.0100	0.0025
$\bar{r}$	0.0109	0.0109	0.0109	0.0109	0.0109	0.0046
RMSE	4.41 · 10^−4^	4.41 · 10^−4^	4.41 · 10^−4^	4.41 · 10^−4^	4.41 · 10^−4^	0.0010
	*W*_11_			*W*_21_		
Truth	0.0952			0.0476		
$\underline{r}$	0.0697			0.0255		
Mean	0.0925			0.0465		
$\bar{r}$	0.1250			0.0634		
RMSE	0.0147			0.0100		
	${\bar{P}}_{11}$			${\bar{P}}_{22}$		
Truth	0.0011			5.13 · 10^−4^		
$\underline{r}$	8.47 · 10^−4^			2.82 · 10^−4^		
Mean	0.0011			5.13 · 10^−4^		
$\bar{r}$	0.0013			8.72 · 10^−4^		
RMSE	1.26 · 10^−4^			1.60 · 10^−4^		

**TABLE 4.** Monte Carlo Simulation for Case 2 with *M* = 100 and PI = 2*σ* (100 Runs).

	*R*					*Q*
	R1	R2	R3	R4	R5	
Truth	1.00	1.00	1.00	1.00	1.00	1.00
$\underline{r}$	0.56	0.56	0.56	0.56	0.56	0.78
Mean	1.05	1.05	1.05	1.05	1.05	0.97
$\bar{r}$	1.51	1.51	1.51	1.51	1.51	1.18
RMSE	0.25	0.25	0.25	0.25	0.25	0.11
	*W*_1_			*W*_2_		
Truth	0.65			0.09		
$\underline{r}$	0.49			−0.03		
Mean	0.63			0.10		
$\bar{r}$	0.80			0.25		
RMSE	0.08			0.07		
	${\bar{P}}_{11}$			${\bar{P}}_{22}$		
Truth	1.89			0.35		
$\underline{r}$	1.59			0.30		
Mean	1.87			0.35		
$\bar{r}$	2.07			0.39		
RMSE	0.12			0.02		

**TABLE 5.** *W* Estimation Monte Carlo Simulation for Case 3 (100 Runs; 10,000 Samples).

	*W*_11_	*W*_21_	*W*_31_	*W*_41_	*W*_51_	*W*_12_	*W*_22_	*W*_32_	*W*_42_	*W*_52_
Truth	0.95	2.80 · 10^−3^	−2.86	−1.76 · 10^−4^	0.03	0.77	0.34	−1.49	0.25	−0.77
Proposed Method	*W*_11_	*W*_21_	*W*_31_	*W*_41_	*W*_51_	*W*_12_	*W*_22_	*W*_32_	*W*_42_	*W*_52_
$\underline{r}$	0.93	−8.65 · 10^−3^	−2.94	−0.02	0.02	0.71	0.31	−1.57	0.18	−0.84
Mean	0.95	2.52 · 10^−3^	−2.86	5.29 · 10^−4^	0.03	0.77	0.34	−1.50	0.25	−0.76
$\bar{r}$	0.97	0.01	−2.80	0.02	0.05	0.84	0.38	−1.41	0.30	−0.68
RMSE	0.01	5.33 · 10^−3^	0.04	9.31 · 10^−3^	9.60 · 10^−3^	0.03	0.02	0.05	0.03	0.04
Mehra’s Method	*W*_11_	*W*_21_	*W*_31_	*W*_41_	*W*_51_	*W*_12_	*W*_22_	*W*_32_	*W*_42_	*W*_52_
$\underline{r}$	0.92	−0.04	−3.38	−0.07	−0.11	0.18	0.04	−2.61	−0.12	−1.17
Mean	1.01	3.79 · 10^−4^	−3.15	3.59 · 10^−3^	−0.02	0.62	0.31	−1.34	0.28	−0.62
$\bar{r}$	1.08	0.06	−2.83	0.08	0.06	1.11	0.60	0.19	0.80	−0.11
RMSE	0.07	0.03	0.33	0.04	0.07	0.30	0.14	0.79	0.22	0.34
Bélanger’s Method	*W*_11_	*W*_21_	*W*_31_	*W*_41_	*W*_51_	*W*_12_	*W*_22_	*W*_32_	*W*_42_	*W*_52_
$\underline{r}$	0.89	−0.02	−3.02	−0.05	−0.04	0.45	0.13	−2.27	−0.02	−1.15
Mean	0.96	1.46 · 10^−4^	−2.85	3.85 · 10^−3^	0.03	0.77	0.33	−1.44	0.26	−0.77
$\bar{r}$	1.01	0.04	−2.70	0.04	0.10	1.13	0.52	−0.32	0.56	−0.32
RMSE	0.03	0.02	0.09	0.02	0.03	0.17	0.09	0.48	0.14	0.20

**TABLE 6.** *R, Q* and $\bar{P}$ Estimation Monte Carlo Simulation for Case 3 (100 Runs; 10,000 Samples).

Method	*R*		*Q*			$\bar{P}$				
	*r*_1_	*r*_2_	*q*_1_	*q*_2_	*q*_3_	${\bar{P}}_{11}$	${\bar{P}}_{22}$	${\bar{P}}_{33}$	${\bar{P}}_{44}$	${\bar{P}}_{55}$
Truth	1.000	1.000	1.000	1.000	1.000	72.31	1.143	1213	0.932	11.74
Proposed Method	*r*_1_	*r*_2_	*q*_1_	*q*_2_	*q*_3_	${\bar{P}}_{11}$	${\bar{P}}_{22}$	${\bar{P}}_{33}$	${\bar{P}}_{44}$	${\bar{P}}_{55}$
$\underline{r}$	0.043	0.918	0.941	0.662	0.802	66.06	0.930	1141	0.633	9.639
Mean	1.067	1.008	0.998	1.000	0.994	72.36	1.146	1212	0.933	11.72
$\bar{r}$	1.976	1.107	1.058	1.261	1.166	77.97	1.334	1290	1.172	13.76
RMSE	0.554	0.052	0.031	0.170	0.097	2.906	0.106	37.87	0.153	1.083
Mehra’s Method	*r*_1_	*r*_2_	*q*_1_	*q*_2_	*q*_3_	${\bar{P}}_{11}$	${\bar{P}}_{22}$	${\bar{P}}_{33}$	${\bar{P}}_{44}$	${\bar{P}}_{55}$
$\underline{r}$	0.102	0.676	0.995	0.060	0.153	69.01	0.540	1270	0.060	0.791
Mean	1.597	1.024	1.224	1.788	0.989	89.88	1.744	1505	1.715	14.22
$\bar{r}$	3.681	1.199	1.420	4.432	2.240	120.7	4.067	1855	4.138	34.21
RMSE	1.484	0.212	0.251	1.464	0.652	23.38	1.280	330.0	1.419	10.61
Bélanger’s Method	*r*_1_	*r*_2_	*q*_1_	*q*_2_	*q*_3_	${\bar{P}}_{11}$	${\bar{P}}_{22}$	${\bar{P}}_{33}$	${\bar{P}}_{44}$	${\bar{P}}_{55}$
$\underline{r}$	0.0270	0.755	0.885	0.043	0.292	60.37	0.564	1069	0.043	2.507
Mean	1.171	1.008	0.992	1.319	1.117	74.58	1.416	1216	1.238	13.86
$\bar{r}$	2.631	1.254	1.126	3.160	2.198	92.54	2.765	1370	2.902	27.078
RMSE	0.789	0.122	0.064	0.829	0.516	9.461	0.667	81.21	0.764	6.829

**TABLE 7.** *R, Q* and $\bar{P}$ Estimation when Varying the Number of Samples Observed *N*, Monte Carlo Simulation for Case 3 (100 Runs; 1,000–10,000 Samples).

	*R*		*Q*			$\bar{P}$				
	*r*_1_	*r*_2_	*q*_1_	*q*_2_	*q*_3_	${\bar{P}}_{11}$	${\bar{P}}_{22}$	${\bar{P}}_{33}$	${\bar{P}}_{44}$	${\bar{P}}_{55}$
Truth	1.000	1.000	1.000	1.000	1.000	72.31	1.143	1213	0.932	11.74
*N* = 1,000	*r*_1_	*r*_2_	*q*_1_	*q*_2_	*q*_3_	${\bar{P}}_{11}$	${\bar{P}}_{22}$	${\bar{P}}_{33}$	${\bar{P}}_{44}$	${\bar{P}}_{55}$
$\underline{r}$	0.075	0.708	0.715	0.593	0.431	62.97	0.965	855.5	0.579	7.505
Mean	2.246	1.014	0.919	1.343	1.120	73.25	1.396	1139	1.263	13.45
$\bar{r}$	5.221	1.358	1.137	2.847	1.542	89.88	2.204	1336	2.677	20.37
RMSE	2.060	0.182	0.133	0.663	0.308	6.998	0.398	136.6	0.626	3.511
*N* = 2,500	*r*_1_	*r*_2_	*q*_1_	*q*_2_	*q*_3_	${\bar{P}}_{11}$	${\bar{P}}_{22}$	${\bar{P}}_{33}$	${\bar{P}}_{44}$	${\bar{P}}_{55}$
$\underline{r}$	0.041	0.767	0.864	0.635	0.719	64.94	0.941	1071	0.660	8.554
Mean	1.453	1.010	0.977	1.127	1.049	73.24	1.245	1195	1.056	12.49
$\bar{r}$	3.354	1.173	1.103	1.685	1.431	80.49	1.653	1342	1.638	16.44
RMSE	1.076	0.094	0.070	0.302	0.181	4.472	0.203	77.80	0.280	2.088
*N* = 5,000	*r*_1_	*r*_2_	*q*_1_	*q*_2_	*q*_3_	${\bar{P}}_{11}$	${\bar{P}}_{22}$	${\bar{P}}_{33}$	${\bar{P}}_{44}$	${\bar{P}}_{55}$
$\underline{r}$	0.043	0.885	0.919	0.578	0.726	65.55	0.856	1126	0.550	8.864
Mean	1.100	1.011	0.997	1.008	0.981	72.27	1.148	1211	0.942	11.62
$\bar{r}$	2.580	1.161	1.084	1.397	1.265	80.13	1.390	1329	1.293	14.85
RMSE	0.757	0.077	0.045	0.218	0.140	3.692	0.136	52.74	0.197	1.530
*N* = 10,000	*r*_1_	*r*_2_	*q*_1_	*q*_2_	*q*_3_	${\bar{P}}_{11}$	${\bar{P}}_{22}$	${\bar{P}}_{33}$	${\bar{P}}_{44}$	${\bar{P}}_{55}$
$\underline{r}$	0.043	0.918	0.941	0.662	0.802	66.06	0.930	1141	0.633	9.639
Mean	1.067	1.008	0.998	1.000	0.994	72.36	1.146	1212	0.933	11.72
$\bar{r}$	1.976	1.107	1.058	1.261	1.166	77.97	1.334	1290	1.172	13.76
RMSE	0.554	0.052	0.031	0.170	0.097	2.906	0.106	37.87	0.153	1.083

**TABLE 8.** *W* Estimation when Varying the Number of Samples Observed *N*, Monte Carlo Simulation for Case 3 (100 Runs; 1,000–10,000 Samples).

	*W*_11_	*W*_21_	*W*_31_	*W*_41_	*W*_51_	*W*_12_	*W*_22_	*W*_32_	*W*_42_	*W*_52_
Truth	0.95	2.80 · 10^−3^	−2.86	−1.76 · 10^−4^	0.03	0.77	0.34	−1.49	0.25	−0.77
*N* =1,000	*W*_11_	*W*_21_	*W*_31_	*W*_41_	*W*_51_	*W*_12_	*W*_22_	*W*_32_	*W*_42_	*W*_52_
$\underline{r}$	0.90	−0.04	−2.97	−0.05	−0.03	0.56	0.27	−1.64	0.14	−0.95
Mean	0.96	−1.57 · 10^−3^	−2.74	7.53 · 10^−3^	0.04	0.79	0.33	−1.49	0.26	−0.79
$\bar{r}$	1.01	0.03	−2.56	0.08	0.09	1.00	0.41	−1.27	0.44	−0.54
RMSE	0.03	0.02	0.17	0.03	0.03	0.11	0.03	0.11	0.08	0.12
*N* =2,500	*W*_11_	*W*_21_	*W*_31_	*W*_41_	*W*_51_	*W*_12_	*W*_22_	*W*_32_	*W*_42_	*W*_52_
$\underline{r}$	0.92	−0.02	−2.97	−0.03	−6.51 · 10^−3^	0.64	0.28	−1.64	0.14	−0.90
Mean	0.96	8.95 · 10^−4^	−2.82	2.17 · 10^−3^	0.03	0.77	0.33	−1.50	0.25	−0.78
$\bar{r}$	1.01	0.02	−2.62	0.05	0.07	0.95	0.40	−1.31	0.36	−0.59
RMSE	0.02	0.01	0.10	0.02	0.02	0.08	0.03	0.09	0.05	0.08
*N* =5,000	*W*_11_	*W*_21_	*W*_31_	*W*_41_	*W*_51_	*W*_12_	*W*_22_	*W*_32_	*W*_42_	*W*_52_
$\underline{r}$	0.92	−0.02	−2.98	−0.03	8.68 · 10^−3^	0.67	0.30	−1.63	0.15	−0.89
Mean	0.95	2.02 · 10^−3^	−2.85	1.15 · 10^−3^	0.03	0.77	0.34	−1.49	0.25	−0.76
$\bar{r}$	0.98	0.01	−2.77	0.03	0.06	0.90	0.39	−1.32	0.32	−0.64
RMSE	0.02	7.08 · 10^−3^	0.06	0.01	0.01	0.06	0.02	0.08	0.05	0.06
*N* =10,000	*W*_11_	*W*_21_	*W*_31_	*W*_41_	*W*_51_	*W*_12_	*W*_22_	*W*_32_	*W*_42_	*W*_52_
$\underline{r}$	0.93	−8.65 · 10^−3^	−2.94	−0.02	0.02	0.71	0.31	−1.57	0.18	−0.84
Mean	0.95	2.52 · 10^−3^	−2.86	5.29 · 10^−4^	0.03	0.77	0.34	−1.50	0.25	−0.76
$\bar{r}$	0.97	0.01	−2.80	0.02	0.05	0.84	0.38	−1.41	0.30	−0.68
RMSE	0.01	5.33 · 10^−3^	0.04	9.31 · 10^−3^	9.60 · 10^−3^	0.03	0.02	0.05	0.03	0.04

**TABLE 9.** Monte Carlo Simulation for Case 4 with *M* = 100 and PI = 2*σ* (100 Runs; 1,000 Samples).

	*R*	*Q*	*W*_1_	*W*_2_	${\bar{P}}_{11}$	${\bar{P}}_{22}$
Truth	1.00	1.00	0.50	1.01	1.01	4.08
$\underline{r}$	3.27 · 10^−3^	0.26	0.05	0.32	0.27	1.09
Mean	1.04	1.02	0.50	1.00	1.03	4.16
$\bar{r}$	2.05	2.09	1.22	2.00	2.09	8.37
RMSE	0.60	0.53	0.32	0.52	0.53	2.11

**TABLE 10.** Monte Carlo Simulation for Case 5 with *M* = 100 and PI = 2*σ* (100 Runs; 1,000 Samples).

	*R*	*Q*	*W*_1_	*W*_2_	*W*_3_	${\bar{P}}_{11}$	${\bar{P}}_{22}$	${\bar{P}}_{33}$
Truth	0.10	0.50	1.14	2.24	3.39	0.54	2.04	4.69
$\underline{r}$	0.07	0.26	0.54	1.05	1.60	0.29	1.07	2.50
Mean	0.11	0.49	1.05	2.06	3.12	0.52	1.99	4.58
$\bar{r}$	0.17	0.65	1.40	2.76	4.13	0.69	2.65	6.09
RMSE	0.03	0.11	0.27	0.54	0.80	0.11	0.45	1.00

## Footnotes

1 The KF is also the best linear estimation algorithm when the noises are non-Gaussian with known covariances [2].

2 Detectability and stabilizability are all that are needed for a stable Kalman filter (i.e., state observability is *not* needed).

3 See Appendix C for a detailed proof.

4 Detailed steps on the gradient computation are provided in Appendix E.

5 0*R* + *R*0 − *RS*^−1^*R* + *G* = 0

6 The solution via Cholesky decomposition and eigen decomposition or simultaneous diagonalization can be found in Appendix F and Appendix G, respectively.

7 The highest probability interval is, assuming unimodality, the minimum width interval such that the estimates of the parameter within the interval have a specified higher probability density than points outside of the interval.

8 All (**R1–R5**) obtain the same values.

## APPENDIXES

### APPENDIX A STEADY-STATE UPDATED STATE COVARIANCE RICCATI EQUATION

From (6) and (9), we can write the steady-state updated state covariance matrix as

(220) $$\bar{P}=P+WSW^{\prime }=FPF^{\prime }+\Gamma Q\Gamma^{\prime }$$

Thus,

(221) $$P=FPF^{\prime }-WSW^{\prime }+\Gamma Q\Gamma^{\prime }$$

Given (14) and (74), we can rewrite (221) as

(222) $$P=FPF^{\prime }-PH^{\prime }R^{-1}SR^{-1}HP^{\prime }+\Gamma Q\Gamma^{\prime }$$

(223) $$=FPF^{\prime }-PH^{\prime }G^{-1}HP^{\prime }+\Gamma Q\Gamma^{\prime }$$

(224) $$=FPF^{\prime }-PH^{\prime }(R-HPH^{\prime }{)}^{-1}HP^{\prime }+\Gamma Q\Gamma^{\prime }$$

### APPENDIX B PROOF OF NECESSARY AND SUFFICIENT CONDITION FOR IDENTIFIABILITY OF UNKNOWN COVARIANCES

*Proposition 5:* The necessary and sufficient condition to estimate the unknown covariance matrices in a system is directly related to its minimal polynomial of its stable closed-loop filter matrix $\bar{F}$ and a transformation of the innovations based on the coefficients of the minimal polynomial.

*Proof:* To prove necessity, let us assume that *Q* and *R* are uniquely estimable, but I does not have a full column rank. This implies the nullspace of I, N(I), contains nonzero vectors. Consequently, the column vectors of I are dependent and there is an infinite number of *Q* and *R* estimates that satisfy the linear equations, which contradicts our original assumption.

Now, to prove sufficiency, let us assume that I has a full column rank. This implies that N(I) contains only the null vector, and thus, a unique solution exists for the *Q* and *R* concatenated into a column vector in (36). Therefore, *Q* and *R* must be estimable.

### APPENDIX C PROOF OF ESTIMABILITY OF *R*

Without loss of generality, let us assume that *a_m_* ≠ 0 and the closed-loop transition matrix $\bar{F}$ is invertible. Note that *W* should be such that $\bar{F}$ does not correspond to a deadbeat observer (which has no noise assumption) or an observer with zero eigenvalues for $\bar{F}$. Since *R* is assumed to be positive definite, $\bar{F}$ is always invertible [22], [43], [52]. When the Kalman filter gain *W* = 0, it is evident that

(225) $$G_{m}=a_{m}I_{n_{z}}$$

Then,

(226) $$L_{m}=a_{m}R$$

and *R* is clearly identifiable. When *W* is not zero, using (21) in (29), we have

(227) $$G_{m}=a_{m}\left[I_{n_{z}}+H(I_{n_{x}}-WH{)}^{-1}W\right]$$

(228) $$=a_{m}(I_{n_{z}}-HW{)}^{-1}$$

Recall that (*I*_*n*_*z*__ − *HW*) is invertible because it relates the innovations and post-fit residuals (see (66)). So, we have

(229) $$(I_{n_{z}}-HW)\;L_{m}=a_{m}R$$

Thus, *R* is estimable.

### APPENDIX D PROCEDURE TO OBTAIN *W* USING MINIMAL POLYNOMIAL

Let *W_s_* be the suboptimal Kalman filter gain and $\tilde{e}$ be the difference of the state predictions between the optimal and suboptimal filter, that is,

(230) $$\tilde{e}(k+1|k)={\bar{F}}_{s}\tilde{e}(k|k-1)+F(W-W_{s})v(k)$$

where ${\bar{F}}_{s}$ is defined as

(231) $${\bar{F}}_{s}=F(I_{n_{x}}-W_{s}H)$$

We can write the suboptimal innovation *ν_s_*(*k*) in terms of $\tilde{e}(k|k-1)$

(232) $$v_{s}(k)=H\tilde{e}(k|k-1)+v(k)$$

Then, using the minimal polynomial of ${\bar{F}}_{s}$ from (21), *ν_s_*(*k* − *i*) can be written as

(233) vs(k−i)=H[F¯sm−ie~(k−m∣k−m−1)]+[∑l=i+1mF¯sl−i−1F(W−Ws)v(k−l)]+v(k−i)

Let us define *ξ*(*k*) as

(234) $$\xi (k)\;=\sum_{i=0}^{m}a_{i}v_{s}(k-i)\;=\sum_{i=0}^{m}a_{i}\left\{\left[H\sum_{l=i+1}^{m}{\bar{F}}_{s}^{l-i-1}F(W-W_{s})v(k-l)\right]+v(k-i)\right\}$$

(235) $$=\sum_{l=0}^{m}\left[a_{l}I_{n_{z}}+H\sum_{i=0}^{l-1}a_{i}{\bar{F}}_{s}^{l-i-1}F(W-W_{s})\right]v(k-l)$$

(236) $$=\sum_{l=0}^{m}V_{l}\;v(k-l)$$

where

(237) $$V_{l}=a_{l}I_{n_{z}}+H\sum_{i=0}^{l-1}a_{i}{\bar{F}}_{s}^{l-i-1}F(W-W_{s})$$

From (15), we can write (236) in terms of z-transform, that is

(238) $$\xi (z)=\sum_{l=0}^{m}V_{l}z^{-l}v(z)$$

Note that we can write *ξ*(*k*) as a vector auto-regressive process of infinite order (which can be truncated to $M^{th}$ order), that is,

(239) $$\xi (k)=\sum_{j=1}^{\infty }Y_{j}\xi (k-j)+v(k)$$

The z-transform of (239) is,

(240) $$\xi (z)={\left[I_{n_{z}}-\sum_{j=1}^{\infty }Y_{j}z^{-j}\right]}^{-1}v(k)$$

Also, note the relationship between (240) and (238),

(241) $$\left(I_{n_{z}}-\sum_{j=1}^{\infty }Y_{j}z^{-j}\right)\sum_{l=0}^{m}V_{l}z^{-l}=I_{n_{z}}$$

By equating coefficients, we have

$$Y_{j}=V_{j}-\sum_{l=1}^{j-1}Y_{j-l}V_{l}\;\;j=0,1,2,\ldots ,m\;\;=-\sum_{l=1}^{m}Y_{j-l}V_{l}\;\;j=m+1$$

We can truncate the infinite vector auto-regressive model at M≫m, for *i* = 1, 2, …, M,

(242) $$E[\xi (k)\xi (k-i{)}^{\prime }]\;\;=E\left\{\sum_{j=1}^{M}Y_{j}\xi (k-j)\xi (k-i{)}^{\prime }+v(k)\xi (k-i{)}^{\prime }\right\}$$

Then, we obtain the estimates of $\{Y_{i}{\}}_{i=1}^{M}$ by solving

$$\sum_{j=1}^{i}Y_{j}L_{i-j}+\sum_{j=i+1}^{m+i}Y_{j}L_{j-i}^{\prime }=L_{i}\;\;i=1,2,\ldots ,m\;\sum_{j=i-m}^{i}Y_{j}L_{m-j+1}+\sum_{j=i+1}^{m+i}Y_{j}L_{j-i}^{\prime }=0\;\;i=m+1,m+2,\ldots ,M$$

Let $\hat{v}(k)$ be

(243) $$\hat{v}(k)=\xi (k)-\sum_{j=1}^{M}Y_{j}\xi (k-j)$$

and recall (237) and note that

(244) $${\hat{C}}_{l}=H\sum_{i=0}^{l-1}a_{i}{\bar{F}}_{s}^{l-i-1}F$$

where $\left\{{\hat{C}}_{l}\right\}$ are the sample covariance matrices. Then,

(245) $$\text{vec}\left[\xi (k)-\sum_{l=0}^{m}a_{l}\hat{v}(k-l)+\sum_{l=1}^{m}{\hat{C}}_{l}FW_{s}\hat{v}(k-l)\right]\;\;=\left[\sum_{l=0}^{m}\hat{v}(k-l{)}^{\prime }⊗{\hat{C}}_{l}\right]\text{vec}(W)$$

Alternately, *W* can be computed via {*V_l_*}. To compute *V_l_*, we know that

(246) $$V_{0}=I_{n_{z}}$$

(247) $$V_{l}=\sum_{i=0}^{l-1}V_{i}Y_{l-i}\;\;l=1,2,\ldots ,m$$

Recalling (237), we have the following relationship,

(248) $${\tilde{V}}_{l}=V_{l}-a_{l}I_{n_{z}}+{\hat{C}}_{l}W_{s}={\hat{C}}_{l}W\;\;l=1,2,\ldots ,m$$

Then,

(249) $$\text{vec}\left(\left[\begin{array}{c} {\tilde{V}}_{1}\\ {\tilde{V}}_{2}\\ \vdots \\ {\tilde{V}}_{m} \end{array}\right]\right)=\left[\begin{array}{c} I_{n_{z}}⊗{\hat{C}}_{1}\\ I_{n_{z}}⊗{\hat{C}}_{2}\\ \vdots \\ I_{n_{z}}⊗{\hat{C}}_{m} \end{array}\right]\text{vec}\;(W)$$

(250) $$={\hat{C}}_{a}\text{vec}\;(W)$$

where the vec(·) function converts *W* into a column vector as in (35) and ⊗ is the Kronecker product. We can obtain the optimal Kalman filter gain *W* by solving the least squares problem, where a unique solution exists if ${\hat{C}}_{a}$ has full column rank.

### APPENDIX E OBJECTIVE FUNCTION GRADIENT COMPUTATION

Note that Θ(*i*), Ψ, and $W\hat{C}(0)$ are all functions of *W* in (54). Thus,

(251) $$\delta J=\frac{1}{2}\text{trace}\left\{\sum_{i=1}^{M-1}[\delta \Theta (i)\Omega +\Theta (i)\delta \Omega ]\right\}$$

where

(252) $$\Omega =[\Psi -WC(0)]\;E^{2}[\Psi^{\prime }-C(0)W^{\prime }]$$

(253) δΩ=∑i=1M−1{[δΨ−δWC(0)]E2[Ψ′−C(0)W′]}+{[Ψ−WC(0)]E2[δΨ′−C(0)δW′]}

and

(254) ∑i=1M−1δΘ(i)Ω=∑i=1M−1{[F′(Inx−(W+δW)H)′F′]i−1}×H′E2H[F(Inx−(W+δW)H)]i−1×{F−F′(F¯′)i−1H′E2HF¯i−1F}Ω

To first order, (254) can be approximated by

(255) ∑i=1M−1−F′[H′δW′(F¯′)i−2+F¯′H′δW′(F¯′)i−3+][⋯+(F¯′)i−2H′δW′]H′E2HF¯i−1FΩ−F′(F¯′)i−1H′E2H[δWHF¯i−2+F¯δWHF¯i−3+][⋯+F¯i−2δWH]FΩ

Then,

(256) $$\sum_{i=1}^{M-1}\delta \Theta (i)\Omega \;\approx -\sum_{i=1}^{M-1}\sum_{\ell =0}^{i-2}\left[F^{\prime }({\bar{F}}^{\prime }{)}^{\ell }H^{\prime }\delta W^{\prime }({\bar{F}}^{\prime }{)}^{i-2-\ell }\right]H^{\prime }E^{2}H{\bar{F}}^{i-1}F\Omega \;\;+F^{\prime }({\bar{F}}^{\prime }{)}^{i-1}H^{\prime }E^{2}H\left[{\bar{F}}^{r}\delta WH{\bar{F}}^{i-2-\ell }\right]F\Omega$$

So,

(257) $$\frac{1}{2}\text{trace}\left(\sum_{i=1}^{M-1}\delta \Theta (i)\Omega \right)\;=-\text{trace}\left[\delta W^{\prime }\sum_{i=1}^{M-1}\sum_{\ell =0}^{i-2}({\bar{F}}^{\prime }{)}^{i-2-\ell }H^{\prime }E^{2}H{\bar{F}}^{i-1}F\Omega F^{\prime }({\bar{F}}^{\prime }{)}^{\ell }H^{\prime }\right]$$

(258) $$=-\text{trace}\left[\delta W^{\prime }\sum_{i=1}^{M-1}\sum_{\ell =0}^{i-2}({\bar{F}}^{\prime }{)}^{i-2-\ell }H^{\prime }E^{2}C(i)E^{2}C(\ell +1{)}^{\prime }\right]$$

For $\sum_{i=1}^{M-1}\Theta (i)\delta \Omega$, we have

(259) ∑i=1M−1Θ(i)δΩ=∑i=1M−1Θ(i){[δPH′−δWC(0)]E2(Ψ′−C(0)W′)}{−[Ψ−WC(0)]E2[HδP−C(0)δW′]}

where,

(260) $$\delta P=\bar{F}P{\bar{F}}^{\prime }-F\delta W(\Psi^{\prime }-C(0)W^{\prime })F^{\prime }\;\;+F(\Psi -WC(0))\delta W^{\prime }F^{\prime }$$

(261) $$=-\sum_{b=0}^{\infty }{\bar{F}}^{b}[F\delta W(\Psi^{\prime }-C(0)W^{\prime })F^{\prime }\;\;+F(\Psi -WC(0))\delta W^{\prime }F^{\prime }]\;({\bar{F}}^{\prime }{)}^{b}$$

Then,

(262) 12trace(∑i=1M−1Θ(i)δΩ)=trace{−δW′∑i=1M−1Θ(i)[Ψ−WC(0)]E2C^(0)}+12[Θ(i)(Ψ−WC(0))E2H]{[+H′E2(Ψ′−C(0)W′)Θ(i)]δP}

Substituting (261) into (265), we get

(263) $$\frac{1}{2}\text{trace}\left(\sum_{i=1}^{M-1}\Theta (i)\delta \Omega \right)\;\;=-\text{trace}\left\{\delta W^{\prime }\sum_{i=1}^{M-1}\Theta (i)(\Psi -WC(0))E^{2}C(0)\right\}\;\;-\text{trace}\{[F\delta W(\Psi^{\prime }-C(0)W^{\prime })F^{\prime }+F(\Psi -WC(0))\delta W^{\prime }F^{\prime }]Z\}$$

(264) Z=∑b=0∞(F¯′)b[12∑i=1M−1[Θ(i)(Ψ−WC(0))E2H]][[+H′E2(Ψ′−C(0)W′)Θ(i)]]F¯b

We can solve for *Z* via a Lyapunov equation as in (61). Then, by substituting *Z* into (263), we have

(265) $$\frac{1}{2}\text{trace}\left(\sum_{i=1}^{M-1}\Theta (i)\delta \Omega \right)\;=-\text{trace}\left\{-\delta W^{\prime }\left[\sum_{i=1}^{M-1}\Theta (i)XE^{2}C(0)+F^{\prime }ZFX\right]\right\}$$

where *X* can be estimated using (63). Then, by subsituting (258) and (265) into (251), we get (60).

### APPENDIX F CHOLESKY DECOMPOSITION AND EIGEN DECOMPOSITION

To solve for *R* using **R3**, we first perform Cholesky decomposition of *S*^−1^. That is,

(266) $$S^{-1}=LL^{\prime }$$

Then,

(267) $$L^{\prime }RS^{-1}RL=(L^{\prime }RL{)}^{2}=L^{\prime }GL$$

Let us perform eigen decomposition on (267), that is

(268) $$L^{\prime }GL=U\Lambda U^{\prime }$$

Then, we have

(269) $$L^{\prime }RL=U\Lambda^{1∕2}U^{\prime }$$

and *R* can be computed as

(270) $$R=(L^{\prime }{)}^{-1}U\Lambda^{1∕2}U^{\prime }L^{-1}$$

### APPENDIX G SIMULTANEOUS DIAGONALIZATION

To solve for *R* using **R3**, we first perform eigen decomposition on *S*^−1^. That is,

(271) $$S^{-1}=U_{1}\Lambda_{1}U_{1}^{\prime }$$

(272) $$=(U_{1}\Lambda_{1}^{1∕2}U_{1}^{\prime }{)}^{2}$$

Noting that

(273) $$S^{-1∕2}GS^{-1∕2}={\left(S^{-1∕2}RS^{-1∕2}\right)}^{2}$$

we perform another eigen decomposition on $U_{1}\Lambda_{1}^{1∕2}U_{1}^{\prime }GU_{1}\Lambda_{1}^{1∕2}U_{1}^{\prime }$ to get

(274) $$U_{1}\Lambda_{1}^{1∕2}U_{1}^{\prime }GU_{1}\Lambda_{1}^{1∕2}U_{1}^{\prime }=(U_{2}\Lambda_{2}^{1∕2}U_{2}^{\prime }{)}^{2}$$

(275) $$=(U_{1}\Lambda_{1}^{1∕2}U_{1}^{\prime }RU_{1}\Lambda_{1}^{1∕2}U_{1}^{\prime }{)}^{2}$$

and *R* can be computed as

(276) $$R=U_{1}\Lambda_{1}^{-1∕2}U_{1}^{\prime }U_{2}\Lambda_{2}^{1∕2}U_{2}^{\prime }U_{1}\Lambda_{1}^{-1∕2}U_{1}^{\prime }$$
